# Modulation of the substrate specificity of the kinase PDK1 by distinct conformations of the full-length protein*

**DOI:** 10.1126/scisignal.add3184

**Published:** 2023-06-13

**Authors:** Mariana Sacerdoti, Lissy Z.F. Gross, Andrew M. Riley, Karin Zehnder, Abhijeet Ghode, Sebastián Klinke, Ganesh Srinivasan Anand, Kristina Paris, Angelika Winkel, Amanda Herbrand, H. Yasmin Godage, Gyles E. Cozier, Evelyn Süß, Jörg O. Schulze, Daniel Pastor-Flores, Mariela Bollini, María Victoria Cappellari, Dmitri Svergun, Melissa A. Gräwert, Pedro F. Aramendia, Alejandro E. Leroux, Barry V.L. Potter, Carlos J. Camacho, Ricardo M. Biondi

**Affiliations:** 1 Department of Internal Medicine I, Universitätsklinikum Frankfurt, Theodor-Stern-Kai 7, 60590 Frankfurt; 2 DKTK German Cancer Consortium (DKTK), Frankfurt, Germany; German Cancer Research Center (DKFZ), D-69120 Heidelberg, Germany; 3 Instituto de Investigación en Biomedicina de Buenos Aires (IBioBA) - CONICET - Partner Institute of the Max Planck Society, Buenos Aires C1425FQD, Argentina; 4 Centro de Investigaciones en Bionanociencias ‘Elizabeth Jares-Erijman’ CIBION. CONICET, Buenos Aires C1425FQD, Argentina; 5 Department of Computational and Systems Biology, University of Pittsburgh, Pittsburgh, PA, 15260, United States of America; 6 Medicinal Chemistry and Drug Discovery, Department of Pharmacology, University of Oxford, Mansfield Road, Oxford, OX1 3QT, U.K; 7 Wolfson Laboratory of Medicinal Chemistry, Department of Life Sciences, University of Bath, Claverton Down, Bath, BA2 7AY, UK; 8 Fundación Instituto Leloir, IIBBA-CONICET, and Plataforma Argentina de Biología Estructural y Metabolómica PLABEM, Buenos Aires C1405BWE, Argentina; 9 Biological Sciences, National University of Singapore,119077, Singapore; 10 Departamento de Química Inorgánica, Analítica y Química Física. FCEN. Universidad de Buenos Aires, Buenos Aires C1428EHA, Argentina; 11 European Molecular Biology Laboratory (EMBL), Hamburg Unit, 22607, Hamburg, Germany; 12 Department of Statistics, University of Pittsburgh, Pittsburgh, PA, WWPH 1821, 15213, United States of America; 13 KBI Biopharma, Technologielaan 8, B-3001 Leuven, Belgium; 14 Department of Chemistry, Huck Institutes of the Life Sciences, The Pennsylvania State University, 104 Chemistry Building, University Park, PA 16802, USA

## Abstract

The activation of at least 23 different mammalian kinases requires the phosphorylation of their hydrophobic motifs by the kinase PDK1. A linker connects the phosphoinositide-binding PH domain to the catalytic domain, which contains a docking site for substrates called the PIF pocket. Here, we used a chemical biology approach to show that PDK1 existed in equilibrium between at least three distinct conformations with differing substrate specificities. The inositol polyphosphate derivative HYG8 bound to the PH domain and disrupted PDK1 dimerization by stabilizing a monomeric conformation in which the PH domain associated with the catalytic domain and the PIF pocket was accessible. In the absence of lipids, HYG8 potently inhibited the phosphorylation of Akt (also termed PKB) but did not affect the intrinsic activity of PDK1 or the phosphorylation of SGK, which requires docking to the PIF pocket. In contrast, the small molecule valsartan bound to the PIF pocket and stabilized a second distinct monomeric conformation. Our study reveals dynamic conformations of full-length PDK1 in which the location of the linker and the PH domain relative to the catalytic domain determines the selective phosphorylation of PDK1 substrates. The study further suggests new approaches for the design of drugs to selectively modulate signaling downstream of PDK1.

## Introduction

Protein kinases play important roles in the regulation of cells and organisms (1). The >500 protein kinases in humans (2) have evolved sophisticated mechanisms of regulation (3, 4) to achieve both selective functions and to phosphorylate substrates in a timely and specific manner (5–7). Because cancer and other diseases often involve altered signaling, protein kinases have become a major drug target group during the last two decades (8). Although most past drug development programs have focused on drugs that bind at the ATP-binding site, there is increased interest in the discovery and development of drugs targeting alternative non-conserved sites on protein kinases, such as by targeting specific allosteric mechanisms of regulation (9–11). Allosteric drug development approaches, however, are hampered by limited information on the dynamic conformations of full-length protein kinases in solution (12).

Phosphoinositide-dependent protein kinase 1 (PDK1) is a master kinase that phosphorylates at least 23 AGC kinases at the activation loop (13, 14), a site required for their activity. Despite its name, PDK1 does not require phosphoinositides for activity (15). The phosphorylation of substrates such as p70 ribosomal S6-kinase (S6K) (16), serum and glucocorticoid-stimulated kinase (SGK) (17) or peptide substrates (such as T308tide (18)) are not affected by phosphoinositides in vitro. Different substrates of PDK1 are involved in different signaling pathways and therefore PDK1 has acquired specific mechanisms for their phosphorylation (19, 20). PDK1 plays a central role in signaling downstream of insulin and growth factors and phosphoinositide 3-kinase (PI3K) (21, 22). The lipid second messenger product of PI3K, phosphatidylinositol 3,4,5-trisphosphate (PIP_3_) is constitutively elevated in most cancer cells, resulting in enhancement of downstream signaling. Indeed, 38% of all cancer patients have loss of PTEN, a key PIP_3_ phosphatase and tumor suppressor, or activating mutations in PI3K (21) which contribute to increased levels of PIP_3_. Thus, members of the growth factor/PI3K pathway are desirable drug targets for the treatment of cancer. Indeed, there are drugs already approved for human use and in clinical development directed at growth factor receptors, PI3K and Akt (also known as protein kinase B; PKB) (21), the best characterized anti-cancer target downstream of PDK1.

**Fig. 1:**
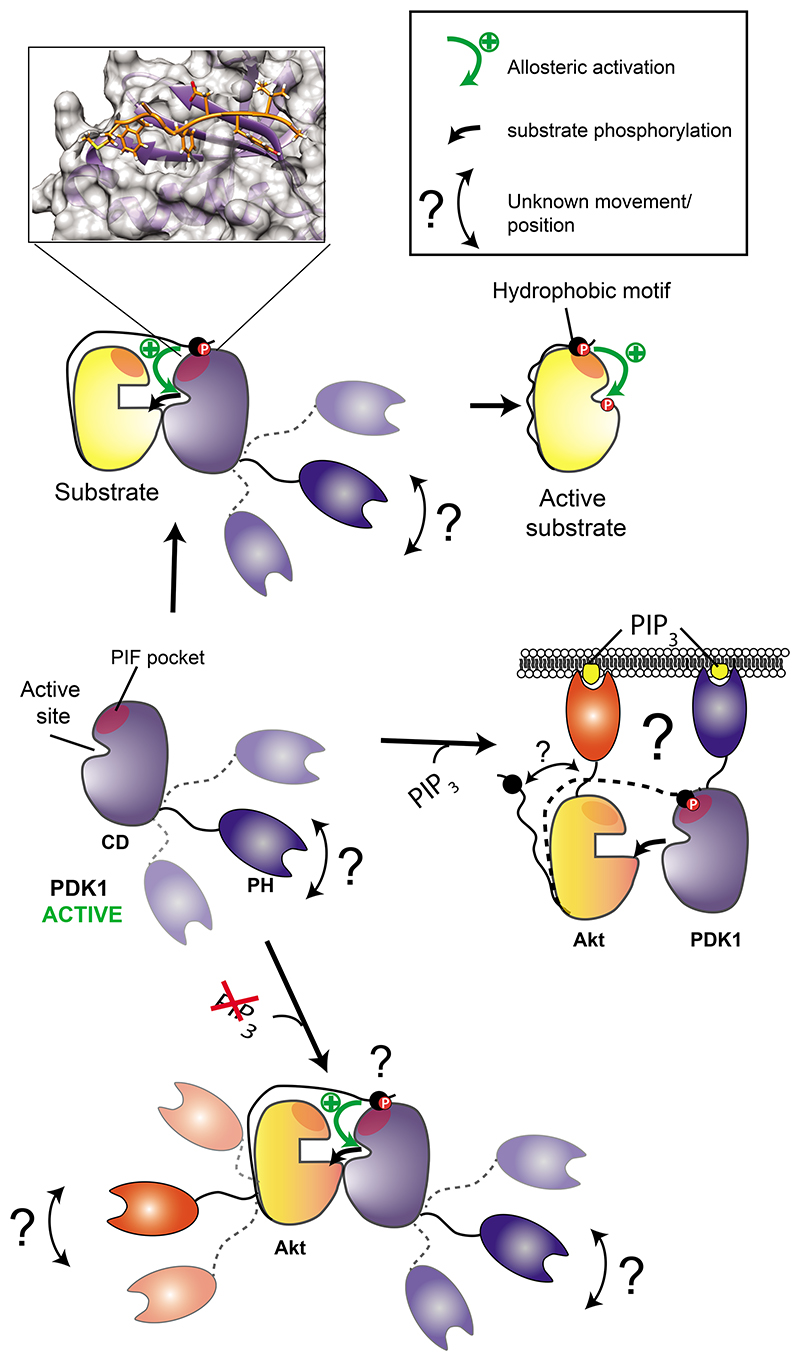
PDK1 phosphorylates substrates by different mechanisms. The phosphorylation of substrates by PDK is regulated by the ability of substrates to interact with PDK1. (Top) Substrates such as S6K or SGK are phosphorylated and activated in vitro in a manner that does not require PIP_3_ or the PH domain of PDK1. Phosphorylation of a hydrophobic motif (HM) on the substrate triggers the docking interaction with the PIF pocket and a neighbouring phosphate-binding site on the small lobe of the catalytic domain of PDK1 (24, 97, 98). The inset shows the polypeptide PIFtide (derived from the HM of the protein kinase C-related protein kinase PRK2) in complex with the PIF pocket of PDK1 (PDB 4RRV) (31). Substrates of PDK1 phosphorylated at the activation loop achieve their active conformation, in which the C-terminal HM binds intramolecularly to the equivalent PIF pocket site on the substrate. (Middle) The phosphorylation of Akt downstream of PI3K requires the co-localization of PDK1 and Akt with PIP_3_, which interacts with the N-terminal PH domain on Akt and the C-terminal PH domain in PDK1 at the cell membrane. (Bottom) Akt can also be phosphorylated by PDK1 in the absence of PIP_3_ (38) and therefore a different mechanism for their interaction should be possible. Although a role of the PH domain of PDK1 in the PIP_3_-dependent phosphorylation of Akt is established (middle), the involvement of the PH domain of PDK1 in the phosphorylation of other substrates is unknown.

Downstream of PI3K activation within the growth factor signaling pathway, PDK1 phosphorylates isoforms of Akt, SGK, RSK and S6K. The phosphorylation of PDK1 substrates follows different mechanisms (Fig. 1). PDK1 does not require binding to PIP_3_ to phosphorylate substrates such as S6K and SGK in vitro or in vivo (16, 17, 23). These phosphorylation events are mediated by a phosphorylation-dependent docking interaction between a C-terminal hydrophobic motif (HM) present in substrates and a regulatory site called the PIF-binding pocket (PIF pocket) in PDK1 (18, 24–33) (Fig. 1, top). On the other hand, the PI3K-induced phosphorylation of Akt requires the co-localization of PDK1 and Akt with PIP_3_ (34), which specifically interacts with an N-terminal PH domain on Akt and a C-terminal PH domain in PDK1 (19, 35) (Fig. 1, middle). Akt lacking its PH domain (Akt ΔPH) resembles SGK but does not become phosphorylated at the activation loop when its C-terminal hydrophobic motif region is replaced by that of SGK (containing the phosphomimetic mutation S422D at the HM site), indicating the existence of additional interaction determinants with PDK1 that differ between SGK and Akt (24).

Although the phosphorylation of some substrates of PDK1 are induced upon PI3K stimulation, other substrates, such as conventional PKCs, are phosphorylated by PDK1 upon synthesis by the HM docking-mediated interaction (36) in a PIP_3_-independent manner (37). Furthermore, persistent inhibition of PI3K can activate Akt by a mechanism in which PDK1 phosphorylates Akt in the absence of PIP_3_ (38). Thus, there are at least two different mechanisms by which PDK1 recognizes its substrates, of which the structural details are known only for the docking interaction of the catalytic domain of PDK1 with the C-terminal region of substrates (Fig. 1, top). Details of the mechanism of interaction and phosphorylation of Akt in the presence and absence of PIP_3_ are unknown (Fig. 1, bottom).

Based on the available biochemical information, the co-localization of PDK1 and Akt by their interaction with PIP_3_ at membranes is thought to determine Akt phosphorylation after PI3K activation. In the presence of PIP_3_-loaded vesicles, the soluble inositol phosphates inositol-1,3,4,5,6-pentaphosphate (IP5) and inositol hexakisphosphate (IP_6_) displace the binding of PIP_3_ to the PH domain of PDK1 and, as expected, inhibit the phosphorylation of Akt by PDK1 (39). IP_5_ (40, 41) and an IP_5_ derivative 2-*O*-benzyl-IP_5_ (42–44) inhibit proliferation and PDK1 activity measured in vitro in the absence of PIP_3_, leading to the suggestion that these compounds inhibit proliferation by inhibition of PDK1-Akt signaling (42).

The existence of PDK1 dimers in cells adds yet another level of regulation (45–48). The yeast PDK1 ortholog PKH2 additionally binds phosphatidic acid, phosphatidylserine and sulfatide, which could participate in its regulation (49). Also, phosphatidylserine regulates the activity and dimerization of PDK1 (50). Analysis of PDK1 interaction with PIP_3_ and phosphatidylserine and its membrane localization and dimerization has not established the roles of monomers and dimers of PDK1 (50). Therefore, even if the current knowledge of PDK1 regulation has allowed us to understand the molecular mechanisms of PDK1 signaling in evolutionarily distant eukaryotic organisms, we still lack information on PDK1 monomers and dimers to help build a complete molecular mechanism of signaling by mammalian PDK1. Autoinhibition of PDK1 mediated by the PH domain and release of the autoinhibition by PIP_3_ has been proposed (51, 52), but should be regarded with caution because constructs of PDK1 without the PH domain have similar catalytic activity as full-length PDK1 (28, 51, 53).

Protein kinases are often multi-domain proteins, regulated by regions N- or C-terminal to their catalytic domains. Although the structures of the catalytic domains of numerous protein kinases are known, the structures of full-length protein kinases and the dynamic conformational changes they undergo are much less studied. The structure of the isolated PH domain of PDK1 and the structure and dynamics of the isolated catalytic domain have been described, but information on the structure and conformations of full-length PDK1 and how PDK1 interacts with Akt are missing. In the current work, we described the existence of at least three distinct conformations of full-length PDK1 in solution in which IP_5_ derivatives and valsartan inhibited the formation of PDK1 dimers by stabilizing two distinct monomeric forms of PDK1. Our studies showed the existence and the structure of a conformation of monomeric PDK1 with the PH domain attached at the back of the catalytic domain that could specifically inhibit the ability of PDK1 to phosphorylate Akt in the absence of PIP_3_ without affecting its ability to phosphorylate SGK. The conformations of full-length PDK1 could be experimentally manipulated and affected substrate selectivity. Together, our results provide insight into the dynamic equilibrium of conformations of full-length PDK1 that adds a missing link into the model of PDK1-specific signaling. Furthermore, the work enables a new approach for discovery of innovative drugs with potential anti-cancer applications.

## Results

### Inositol phosphate derivatives potently inhibited the phosphorylation of Akt by PDK1 but did not inhibit PDK1 kinase activity towards all polypeptide substrates

**Fig. 2:**
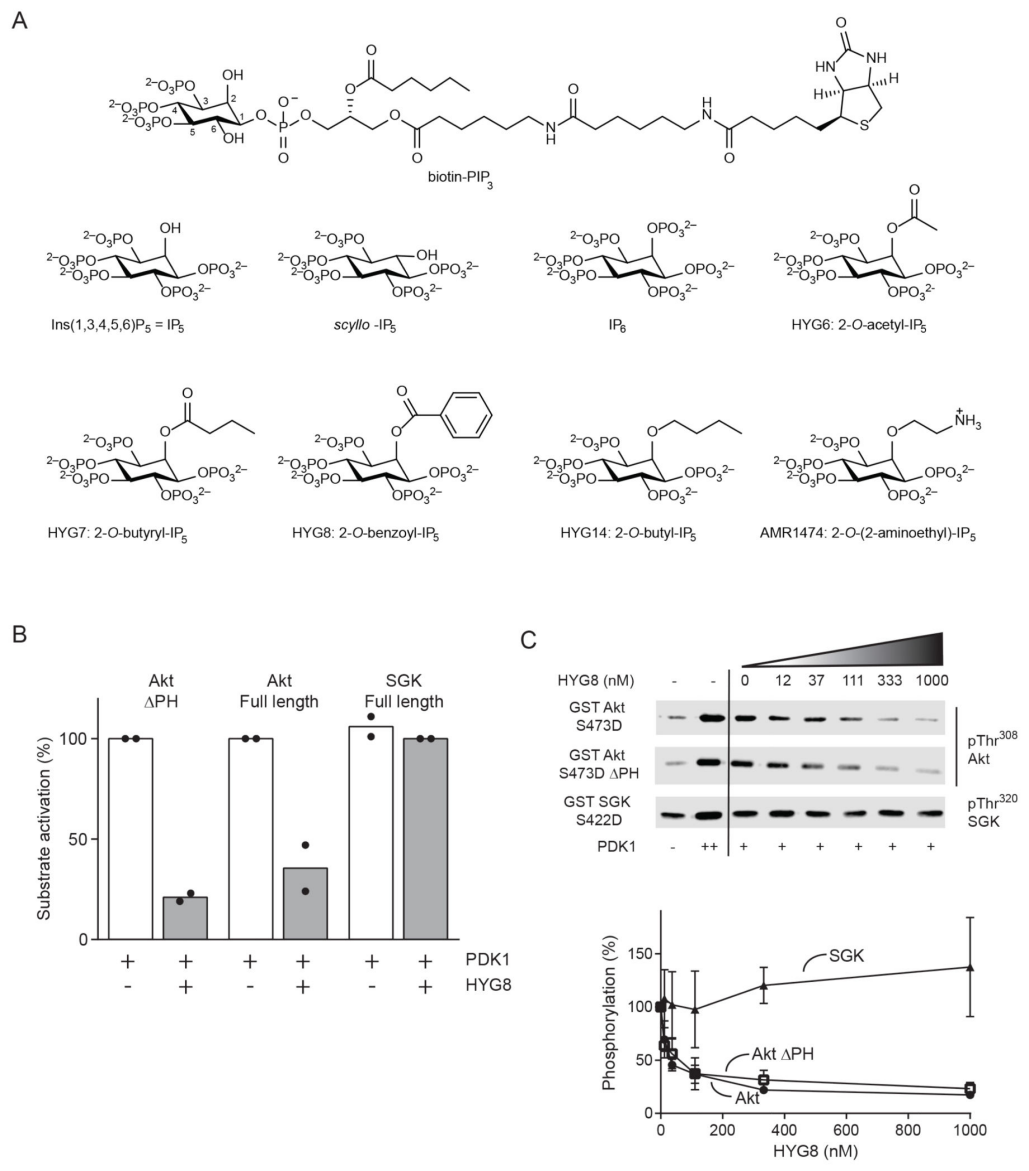
HYG8 inhibits the PDK1-mediated activation of Akt but not that of SGK. (A) Chemical structures of biotin-PIP_3_ and the inositol phosphates and derivatives used. (B) Effect of HYG8 (1 μM) on the PDK1-mediated activation of the Akt ΔPH [S473D] construct lacking the PH domain, full-length Akt [S473D], and full-length SGK [S422D]. The activity of PDK1_1-556_ was measured indirectly by its ability to activate GST-Akt [S473D] and GST-SGK [S422D]. The activity of Akt and SGK were measured using [γ^32^P]ATP and KK-Crosstide as substrates. The HM phosphorylation sites in GST-Akt [S473D] and GST-SGK [S422D] are mutated to Asp. Before the assay, the two substrates were dephosphorylated in vitro to ensure that the activity of the Akt and SGK constructs depended only on the phosphorylation of their activation loops by PDK1. N=2 independent experiments. (C) In vitro substrate phosphorylation by PDK1_1-556_ was evaluated in the presence of increasing HYG8 concentrations using phospho-specific antibodies. Lane 1 shows basal phosphorylation; lane 2 shows phosphorylation in the presence of excess PDK1; lanes 3-8 show phosphorylation by PDK1 with increasing HYG8 concentrations. Quantification of fluorescence from Western blots is shown. N=4 independent experiments. One representative Western blot is shown.

2-*O*-benzyl-IP_5_ is a potent in vitro inhibitor of PDK1 (IC_50_ 26 nM) (42). To explore the structural basis of this effect, we synthesized additional inositol phosphate derivatives related to IP_5_ (Fig. 2A). We found that the synthetic IP_5_ derivative 2-*O*-benzoyl-IP_5_ (HYG8) was also a potent inhibitor of PDK1, as measured by two different in vitro commercial kinase assays performed in the absence of PIP_3_. The first was Thermo Fisher Scientific/Invitrogen PDK1 cascade assay, which uses Akt as substrate in a coupled, cascade-type assay and in which the activity of PDK1 is measured by its ability to activate Akt. The second was the “PDK1 Direct” assay, which uses a peptide substrate (Ser/Thr 07) of undisclosed sequence (table S1) as the substrate of PDK1 and a readout of FRET between coumarin and fluorescein (5-FAM). In the PDK1 Direct assay, HYG7, HYG8 and HYG14 inhibited PDK1 with IC_50_ values of 16-29 nM range (table S1), whereas IP_5_ and 2-*O*-acetyl-IP_5_ (HYG6) were 15 to 36 times less potent inhibitors. IP_6_ was inactive. *Scyllo*-IP_5_ and another synthetic derivative of IP_5_, 2-*O*-(2-aminoethyl)-IP_5_ (AMR1474), were weak inhibitors in the same assays (10% or less inhibition at 1 μM) (table S2).

We have previously measured the intrinsic activity and allosteric activation of PDK1 by measuring the ability of PDK1 to phosphorylate a peptide substrate (T308tide, KTFCGTPEYLAPEVRR) derived from the site in Akt that is phosphorylated by PDK1 (18, 28, 29, 54). In our established assay, the HYG compounds were not potent inhibitors of PDK1, although HYG8 partially inhibited full-length PDK1, but not the isolated catalytic domain (fig. S1A,B). Thus, results using our established in vitro assay contrasted with those from commercial assays reported previously for 2-*O*-benzyl-IP_5_ and described above, in which the related compounds HYG7, HYG8 and HYG14 potently inhibited the kinase activity of PDK1. This finding suggested that interactions with the PH domain have an unexpected effect on PDK1 activity in an assay-dependent manner.

### Inositol phosphates and synthetic derivatives inhibited the PDK1-mediated phosphorylation and activation of Akt but not of SGK in vitro

In previous studies, we concluded that the structural determinants used by PDK1 for the phosphorylation of Akt should be different from that of SGK (24). We and others also have described small molecules that bind to the PIF pocket of PDK1 and inhibit the phosphorylation of SGK and S6K but not that of Akt (29, 31). PDK1 can activate Akt and Akt ΔPH in vitro in the absence of PIP_3_, although with lower efficiency, in a mechanism that depends on the HM-mediated docking interaction(24). We found that in the absence of PIP_3_, HYG8 selectively inhibited the activation of Akt and Akt ΔPH by PDK1, but did not inhibit the activation of SGK in vitro (Fig. 2B,C). The inhibition of Akt activation by HYG8 replicated the commercial “cascade” PDK1 assay, validating some of the results described above. In addition, the lack of effect on SGK was consistent with our finding that the HYG compounds did not inhibit the intrinsic activity of PDK1 (fig. S1 A,B). Moreover, the data indicated that the interaction of PDK1 with HYG8 promoted conformational changes that selectively blocked the phosphorylation and activation of Akt, without affecting the phosphorylation of SGK.

We explored the possible reason for the different results observed with the commercial PDK1 Direct assay and our established activity assay using T308tide as a substrate. We showed that the phosphorylation of a synthetic peptide substrate of PDK1 was sensitive to the presence of the linker-PH domain and that the interaction with this peptide substrate was inhibited by HYG8 (fig. S2, A to G). In turn, our results suggested that the conformational change induced by HYG8 could also affect the phosphorylation of Ser/Thr 07, a structurally related substrate peptide used in the commercial “PDK1 Direct” assay. Together, our results indicated that small molecules such as HYG8 could selectively inhibit the activation of Akt by PDK1.

### Inositol phosphates and synthetic derivatives bound differentially to the isolated PH domain of PDK1 and to full-length PDK1

The compounds in Fig. 2A bound to the isolated PH domain of PDK1 as shown by their ability to displace the interaction of the isolated PH domain of PDK1 (GST-PDK1_408-556_) and full-length PDK1 (GST- PDK1_1-556_) from membranes spotted with di-C_18_-PIP_3_ in a lipid overlay assay. The effect of the different compounds on the interaction depended on the PDK1 construct (fig. S3A). For example, IP_5_, *scyllo*-IP_5_, IP_6_ and HYG14 displaced the interaction of PIP_3_ with the isolated PH domain to a similar extent, but HYG14 was much more potent in displacing the interaction between PIP_3_ and full-length PDK1_1-556_.

**Fig. 3:**
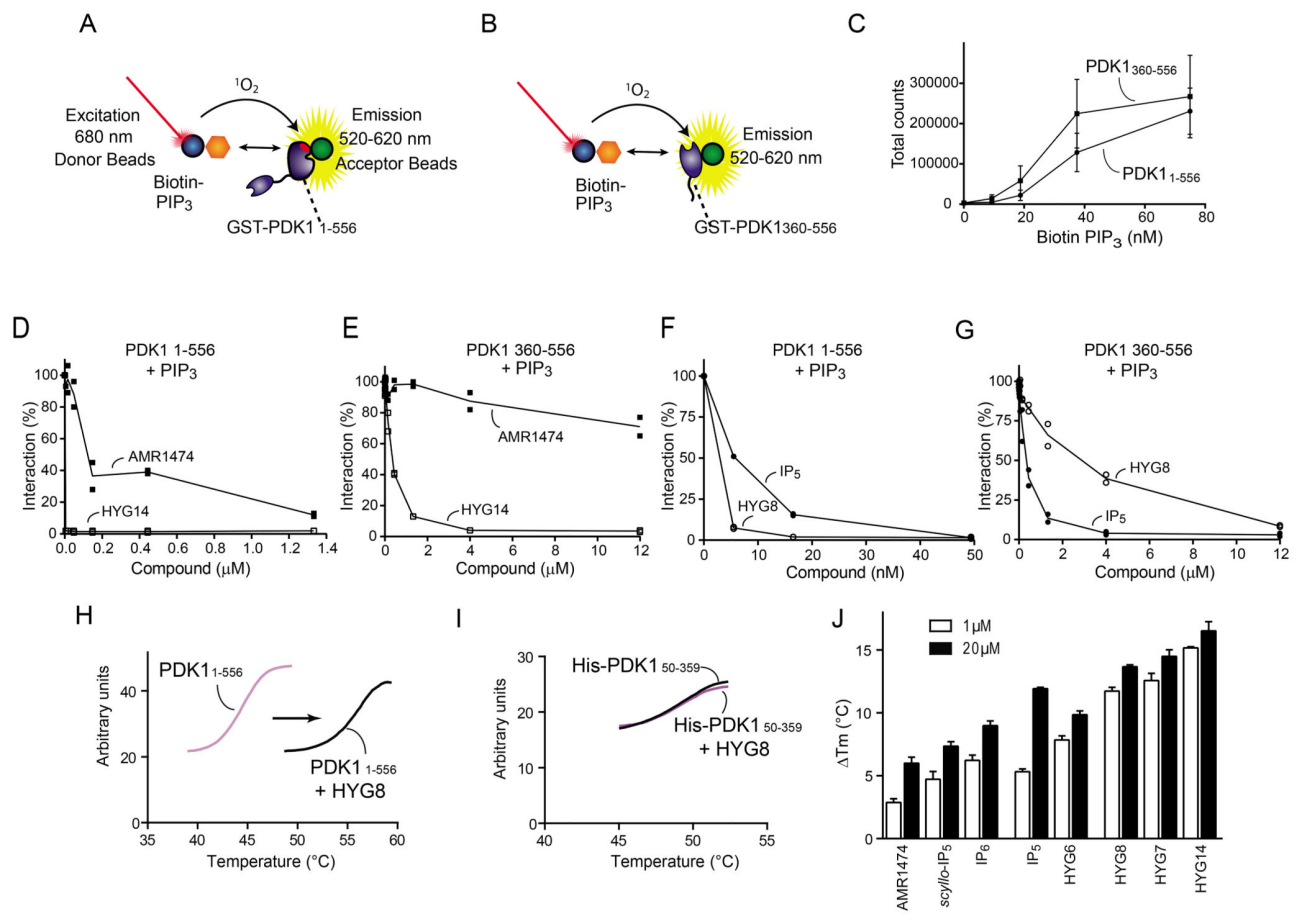
Inositol phosphates and synthetic derivatives differentially displace PIP_3_ from PDK1_1-556_ and from the PH domain of PDK1 and thermally stabilize PDK1_1-556_ to differing extents. (A,B) Schematic representations of the AlphaScreen interaction assays. Interaction of biotin-PIP_3_ with GST-PDK1_1-556_ (A) and with GST-PDK1_360-556_, which includes only the linker and PH domain of PDK1 (B). (C) AlphaScreen interaction between biotin-PIP_3_ and GST-PDK1_360-556_ (4 nM) or GST-PDK1_1-556_ (4 nM). N=3 independent experiments. (D-G) Effect of HYG14 and AMR1474 on the interaction of PIP_3_ (20 nM) with GST-PDK1_1-556_ (D) or GST-PDK1_360-556_ (E). Effect of HYG8 and IP_5_ on the interaction of PIP_3_ (20 nM)with GST-PDK1_1-556_ (F) or GST-PDK1_360-556_ (G). N=2 independent experiments. GST-PDK1 constructs were used at 3 nM. (H-J) Differential scanning fluorimetry was used to assess the effect of HYG8 on the thermal stability (ΔTm) of PDK1_1-556_ (H) and PDK1_50-359_ (I) and of the indicated inositol phosphates and derivatives at 1 μM (white) or 20 μM (black) on PDK1_1-556_ (J). N=3 independent experiments.

We established an AlphaScreen assay that measured the interaction between biotin-PIP_3_ and GST-PDK1_1-556_ or GST-PDK1_360-556_ (GST-linker-PH domain) (Fig. 3A,B). Similar to the results obtained in the lipid overlay assay, both proteins interacted equally well with biotin-PIP_3_ (Fig. 3C), but the ability of the compounds to displace the PDK1-PIP_3_ interaction depended on the construct. For example, HYG14 almost completely displaced PIP_3_ from GST-PDK1_1-556_ at 5 nM (Fig. 3D, fig. S3B) but did not achieve similar displacement of PIP_3_ from GST-linker-PH domain unless added at a more than 1000-fold higher concentration (Fig. 3E, fig. S3B). In contrast, 2-*O*-aminoethyl-IP_5_ (AMR1474), a HYG14 derivative with an amino group at the end of the hydrophobic side chain, lacked or showed reduced ability to displace the interaction of PIP_3_ from GST-PDK1_1-556_ and GST-PDK1_360-556_ (Fig. 3D,E, fig. S3B). Similarly, HYG8 displaced PIP_3_ from GST-PDK1_1-556_ better than from GST-PDK1_360-556_ and showed a higher potency than IP_5_ in the first assay, showing again the importance of a hydrophobic side chain on full-length PDK1 (Fig. 3F,G, fig. S3B). Together, the results suggested that HYG7, HYG8 and HYG14 interacted with the PH domain of PDK1 but promoted additional interactions with regions outside the PH domain that provided higher affinity for full-length PDK1.

We next investigated the effect of the different compounds on the thermal stability of the kinase. HYG8 increased the temperature stability of PDK1_1-556_ without affecting that of PDK1_50-359_, which comprises only the catalytic domain (Fig. 3H,I). HYG8 also stabilized GST-PDK1_1-556_ (fig. S4), indicating that the stabilization was independent of the tag. In a manner that mirrored the relative potency to displace PIP_3_ from PDK1_1-556_ and the effect on PDK1 activity in the commercial PDK1 Direct assay, the relative potency of the different compounds could be divided into three groups. HYG7, HYG8 and HYG14 at 1 μM potently increased the thermal stability of PDK1_1-556_ (1 μM) by 11-15 °C, while they produced a minor further increase in the temperature stability (13-16 °C) at 20 μM. The stabilization at both temperatures was lower by AMR1474 and *scyllo*-IP_5_, while IP_5_ and HYG6 had intermediate potency. (Fig. 3J and table S3).

### Inositol phosphates and synthetic derivatives stabilized a particular conformation of full-length PDK1

**Fig. 4:**
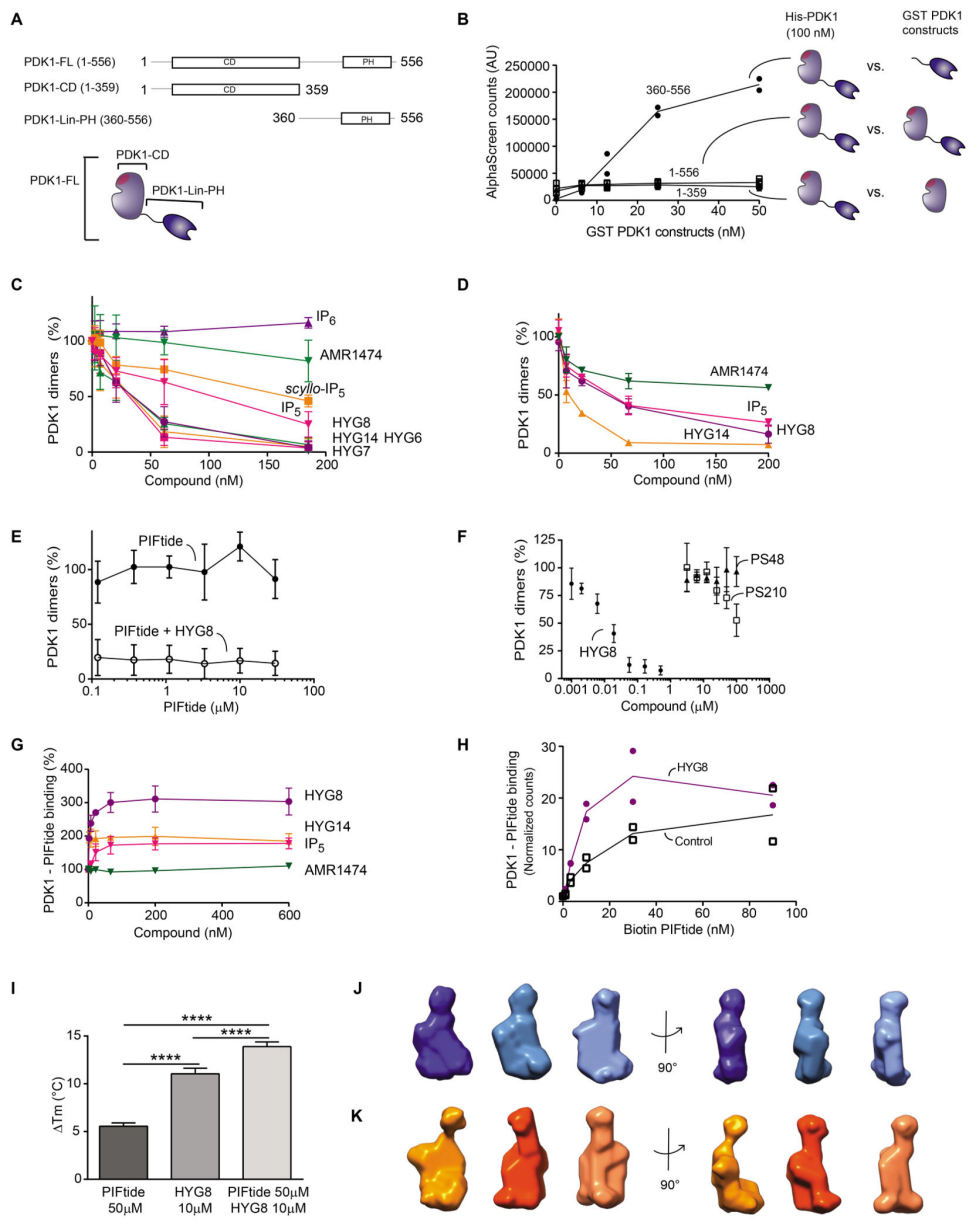
Inositol phosphates and synthetic derivatives inhibit the formation of PDK1 dimers. (A) Schematic representation of constructs used: full-length PDK1 (PDK1_1-556_), the catalytic domain of PDK1 (PDK1_50-359_) and the C-terminal construct comprising the linker region and the PH domain (PDK1360-556). (B) AlphaScreen interaction assay to measure the formation of PDK1 dimers. N=2 independent experiments. (C,D) Effect of inositol phosphates and derivatives on the interaction between HisPDK1_1-556_ and GST-PDK1_360-556_ (C) and on the interaction between His-PDK1_1-556_ and GST-PDK1_1-556_ (D). N=3 independent experiments. (E) Effect of PIFtide on the dimer interaction between His-PDK1_1-556_ and GST-PDK1_360-556_ and the dimerization displacement by HYG8 (500 nM). N=3 independent experiments. (F). Effect of PS210 and PS48 on the dimer interaction between His-PDK1_1-556_ and GST-PDK1_360-556_. N=3 independent experiments. (G) Interaction of PDK1 with PIFtide and effect of HYG8, HYG14, IP_5_, or AMR1474 on the PDK1-PIFtide interaction. N=3 independent experiments.(H) The effect of the presence or absence of HYG8 (300 nM) on the PDK1-PIFtide interaction. N=2 independent experiments. (I) Differential scanning fluorimetry was used to assess the effect of PIFtide and HYG8 individually or together on the thermal stability of PDK1_1-556_. N=3 independent experiments for each group. **** p<0,0001 One-way ANOVA followed by Tuckey post hoc test with Bonferroni correction. (J,K) Ab initio low resolution structure determination from experimental SEC-SAXS data using DAMMIF for apo His-PDK1_1-556_ (J) and His-PDK1_1-556_ + HYG8 (K). Out of 10 to 20 runs, the 3 most representative DAMMIF models according to SUPCOMB are depicted.

We reasoned that the increased temperature stability of full-length PDK1 by HYG7, HYG8 and HYG14 could be due to the stabilization of a dimeric form of the kinase or the intramolecular stabilization of the monomeric form. Therefore, we next sought to set-up a reliable PDK1– PDK1 interaction assay (Fig. 4A). His-PDK1_1-556_ interacted with GST- PDK1_1-556_ (Fig. 4B. However, the interaction between His-PDK1_1-556_ and a construct of PDK1 comprising the linker region and the PH domain (GST-PDK1-Linker-PH; GST-PDK1360-556) showed a much higher level of binding (Fig. 4B). We found that HYG6, HYG7, HYG8 and HYG14 potently blocked the formation of PDK1 dimers (IC_50_ 20-35 nM; Fig. 4C and table S4). IP_5_ and *scyllo-IP*_5_ had an intermediate effect (IC_50_ 67 and 124 nM), whereas AMR1474 and IP6 had limited ability to displace the interaction (IC_50_ 470 nM and no displacement, respectively). The effect of compounds on the dimerization assay using full-length His-PDK1_1-556_ and GST-PDK1_1-556_ had similar results (Fig. 4D and fig. S5). The relative ability of compounds to displace the dimer interaction correlated with their ability to displace the binding of PIP_3_ from full-length PDK1 and the potent inhibition of PDK1 activity measured in the commercial assays.

Our data suggest that the phenyl group side chain of HYG8 (or the aliphatic side chain of HYG7 and HYG14) induced the intramolecular interaction of the PH domain with the catalytic domain. The effect was induced most effectively by the phenyl (HYG8) or aliphatic (HYG14) group but not by AMR1474, thus indicating that it was specific and abolished by a positive charge at the end of the hydrophobic chain. The axial orientation of the hydroxyl group in IP_5_ was favored compared with the equatorial orientation in *scyllo*-IP_5_, showing that the effect could be sensitive to very small structural changes in inositol phosphate ligands. Moreover, a phosphate at this position was not favored because IP_6_ was less potent than IP_5_.

### Binding to the inositol polyphosphate compound HYG8 switched the equilibrium of full-length PDK1 to a monomeric form that bound PIFtide

There is an allosteric communication between the PIF pocket regulatory site and the active site in PDK1 (55). PIFtide is the prototype hydrophobic motif (HM) polypeptide derived from the C-terminal HM of a PDK1 substrate. HM polypeptides from substrates of PDK1 interact with the PIF pocket of PDK1, providing selectivity and regulation of the interaction with substrates and also enhancing the catalytic activity of PDK1 (18, 28, 54). PIFtide had no effect on dimer formation (Fig. 4E). Like PIFtide, small compounds that bind to the PIF pocket (PS48 and PS210) allosterically affect the ATP-binding site, whereas some compounds that bind to the ATP-binding site (PS653, adenosine and GSK2334470) allosterically affect the properties of the PIF pocket (29, 54). None of these compounds affected the dimerization of PDK1 (Fig. 4F and fig. S6, A and B). Therefore, we conclude that the allosteric communication between the ATP-binding site and the PIF pocket in the catalytic domain of PDK1 was independent of the dimerization mechanism. We reasoned that if PIFtide bound only dimers or only monomers, we would observe a shift in the monomer-dimer equilibrium, which did not happen. Therefore, we also concluded that PIFtide binds both PDK1 dimers and monomeric conformations of PDK1.

HYG8 increased binding of GST-PDK1_1-556_ to PIFtide 3-4-fold in different experiments with approximate EC_50_ 2.4 nM, whereas AMR1474 had virtually no effect (Fig. 4G). The increased level of binding to PIFtide could be due to an increased affinity for PIFtide. However, the concentration of biotin-PIFtide that produced half maximal binding to PDK1 was similar in the presence or absence of HYG8 (Fig. 4H), suggesting that the affinity for PIFtide was unchanged by HYG8. In any case, an experimental increase in the maximal binding could be only due to a change in the equilibrium of PDK1 conformations, with an increase in the population of PDK1 molecules able to bind to PIFtide. PIFtide and HYG8 produced additive stabilization of PDK1 in a temperature stability assay (Fig. 4I), thus confirming that PIFtide can bind to the HYG8-stabilized conformation of PDK1. Together, these data led us to conclude that HYG8 could increase the number of PDK1 proteins that bound to PIFtide. An increase in PDK1 molecules that bind to PIFtide in the presence of HYG8 must originate from a pre-existing pool of molecules that have reduced or zero ability to bind PIFtide, large enough to account for the observed several fold increase in binding to PIFtide. Therefore, we suggested that in solution, there is an equilibrium between different pools of PDK1 and that the binding of HYG8 shifts the landscape to favour monomeric conformations that bind PIFtide.

In batch small-angle X-ray scattering (SAXS), the estimated Dmax and Rg were decreased in the presence of HYG8, leading to a decrease in the estimation of the MW (from an estimated MW of 85-94 kDa to an estimated MW of 53 kDa; table S5). We also established a STORM-based method to visualize PDK1 dimers. In this assay, we diluted fluorescently labelled SNAP-PDK1 to ensure individual proteins were separated. Because the assay cannot distinguish between the two molecules forming part of the dimer, each PDK1 dimer is expected to appear as a single fluorescent spot with twice the fluorescence intensity of a monomer. A bimodal distribution of fluorescence intensity was identified (fig. S7A), in accordance with the existence of monomers and dimers of PDK1. The addition of HYG8 decreased the proportion of PDK1 dimers (fig. S7B). Together, the results above indicated that PDK1 was in equilibrium between monomers and dimers and that HYG8 disrupted dimers by stabilizing a particular monomeric conformation of PDK1 that could bind PIFtide.

To analyze only the monomeric forms of PDK1, we subjected PDK1_1-556_ to size exclusion chromatography (SEC)-SAXS. Monomeric full-length PDK1_1-556_ had D_max_ = 110 Å and Rg = 34.8 Å, with the MW estimated between 67 and 79 kDa by different calculation approaches (Table 1). When the SEC-SAXS experiment was performed after incubation of full-length PDK1_1-556_ with HYG8 and the inclusion of 1 μM HYG8 in the running buffer, the D_max_ and Rg remained similar (D_max_ = 115 Å and Rg = 35 Å respectively) with an estimated MW by different calculation approaches from 54 to 61 kDa (Table 1 and fig. S8A). Furthermore, we used the SEC-SAXS data with DAMMIF software to build ab initio low resolution 3D models of PDK1_1-556_ in the absence or in the presence of HYG8 (Fig. 4J,K).

The SAXS measurements can be used to model the ensemble of flexible multi-domain proteins in solution using ensemble optimization method software (EOM)(56). In the EOM study, the software first calculates the radius of gyration (Rg) and maximal diameter (D_max_) of an ensemble of full-length PDK1 generated models under the condition that the CD and PH domains are well structured but the linker region can be freely located (fig. S8 B and C, black lines). In contrast to the flexible hypothesis, the selected models that best fitted the experimental data possessed lower Rg and D_max_ values, which indicated that PDK1 was considerably compacted in the apo and HYG8-bound conformations (fig. S8 B and C, red and blue lines). Furthermore, EOM confirmed that the monomeric form of PDK1_1-556_ did not substantially change the level of compactness upon interaction with HYG8. Therefore, we concluded that the linker-PH domain is not freely and randomly mobile, but most of the time specifically attached to the catalytic domain. The majority of the proposed EOM models for full-length PDK1 in the absence of HYG8 present the PH domain in close proximity to the upper part (small lobe) of the catalytic domain (fig. S8D). The PH domain was modeled more frequently on the back side of the catalytic domain in the presence of HYG8 (fig. S8E).

### Confirmation of the second monomeric conformation of PDK1 using valsartan, a small molecule that bound to the PIF pocket and stabilized a different monomeric form of PDK1

**Fig. 5:**
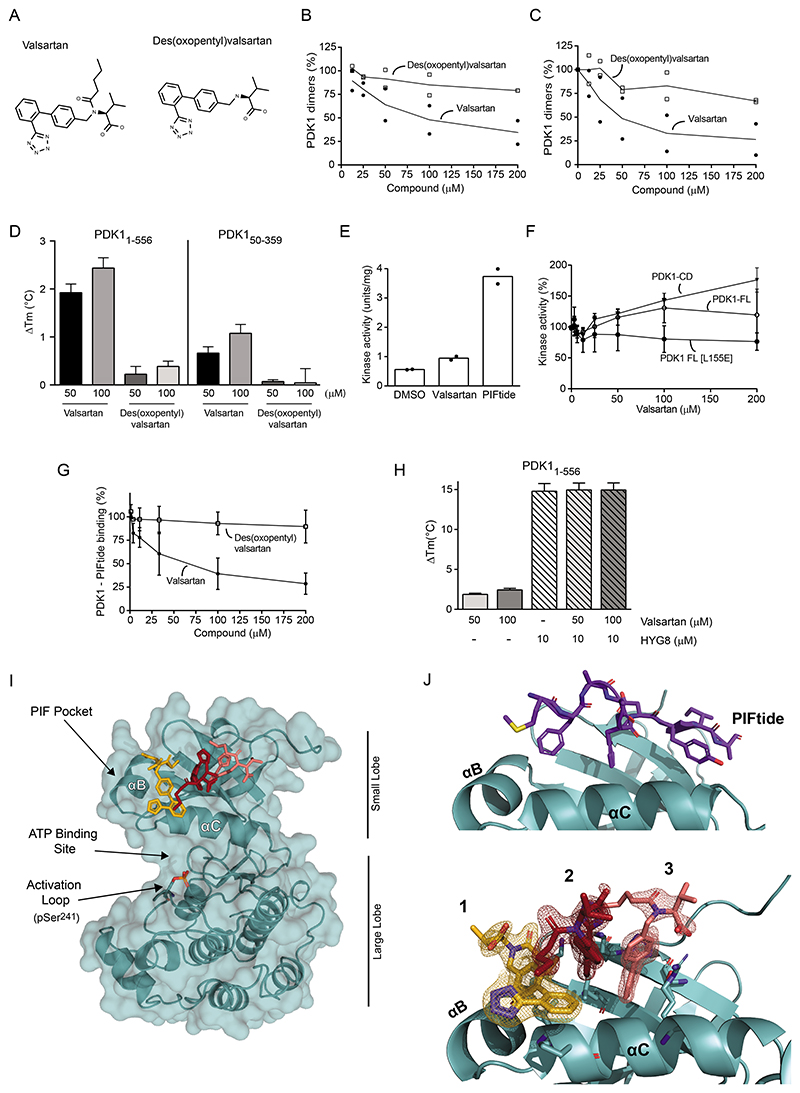
Valsartan inhibits PDK1 dimerization by interacting with the PIF pocket of PDK1. The AlphaScreen PDK1 dimerization assay (His-PDK1_1-556_ and GST-PDK1_360-556_) was used to screen a compound library (Prestwick Chemical Library®) for small molecules that affect the interaction. (A) Chemical structures of the hit compound identified, valsartan, and the inactive related compound, des(oxopentyl) valsartan. (B and C) The effect of valsartan and des(oxopentyl) valsartan on the interaction between His-PDK1_1-556_ and GST-PDK1_1-556_ (B) and on the interaction between His-PDK1_1-556_ and GST-PDK1_360-556_ (C) was assessed. N=2 independent experiments. (D) Thermal stability of PDK1_1-556_ and PDK1_50-359_ in the presence of valsartan and des(oxopentyl) valsartan. N=3 independent experiments. (E) The effect of DMSO, valsartan (200μM) and PIFtide (2μM) on the specific activity of the catalytic domain of PDK1 (PDK11-359). N=2 independent experiments. (F) The effect of valsartan was tested on the activity of the catalytic domain of PDK1 (PDK11-359; catalytic domain, CD), on WT full-length PDK1 (PDK1_1-556_; FL) and full-length PDK1 mutated at a central residue in the PIF pocket, L155E (PDK1 FL [L155E]). N=3 independent experiments. (G) Effect of valsartan and des(oxopentyl) valsartan on the interaction between GST-PDK1_1-556_ and biotin-PIFtide as assessed with AlphaScreen technology. N=3 independent experiments. (H) Effect of valsartan on the thermal stabilization of PDK1_1-556_ by HYG8. N=3 independent experiments. (I) Crystal structure of PDK1 in complex with valsartan (PDB: 8DQT). (J) Magnified view of the PIF pocket of PDK1 depicting the PIFtide binding mode (top) (PDB: 4RRV (31)) and of the same orientation of the pocket in complex with the three molecules of valsartan. The 2*mFo-DFc* electron density map of the valsartan molecules is shown at the σ=1.0 level (bottom).

We showed that HYG8 increased the number of PDK1 molecules that bound to PIFtide (Fig. 4G,H), suggesting the existence of a pool of PDK1 conformations that did not bind to PIFtide. To identify and characterize the predicted second monomeric conformation of PDK1, we screened a small library of drug-like compounds for inhibitors of PDK1 dimerization. We used the AlphaScreen interaction assay that measures the interaction between His-PDK1_1-556_ and GST-PDK1_360-556_ (Fig. 4B), eliminated compounds with non-specific effects, and validated a set of “hits” with IC_50_ in the 2-50 μM range. The angiotensin II type 1 receptor blocker valsartan inhibited PDK1 dimerization (IC_50_= 32 μM, Fig. 5A-C). Valsartan is based on a tetrazole, and is linked to a biphenyl structure, an aminobutyric acid and an oxopentyl side chain. Des(oxopentyl) valsartan did not disrupt PDK1 dimers, suggesting that the oxopentyl moiety was important for the interaction and disruption of PDK1 dimers. At 50 μM, valsartan stabilized PDK1_1-556_ in the temperature-stability assay (ΔTm 2.2 °C; Fig. 5D) but produced less stabilization of the isolated catalytic domain of PDK1 (PDK1_50-359_) (Fig. 5D). The result confirmed that valsartan binds to PDK1_1-556_ and that the higher stabilization requires the linker-PH domain region.

Valsartan modestly increased the ability of PDK1_50-359_ to phosphorylate the T308tide peptide (Fig. 5E) and differentially affected WT PDK1_1-556_ and PDK1_1-556_ [L155E] that is mutated at the PIF pocket (Fig. 5F). Compounds that bind at the PIF pocket - PS48, PS210, and RS1 - allosterically activate PDK1 and increase the phosphorylation of T308tide (29, 31, 54). The effect of valsartan on WT PDK1_1-556_ but not PDK1_1-556_ [L155E] suggested that valsartan binds to the PIF pocket as well. In agreement with this prediction, valsartan but not des(oxypentyl)valsartan inhibited the interaction between His-PDK1_1-556_ and biotin-PIFtide (Fig. 5G). Although PDK1 could bind HYG8 and PIFtide simultaneously, valsartan did not further enhance the temperature stability of the PDK1-HYG8 complex (Fig. 5H). These results are consistent with valsartan stabilizing a monomeric form of full-length PDK1 that is distinct from the monomeric form stabilized by HYG8 and functionally different because it does not bind PIFtide.

To further understand the binding mode of valsartan, we crystallized PDK1_50-359_ in complex with valsartan. We solved the high resolution structure of the PDK1-valsartan complex (Fig. 5I and table S6), which showed that valsartan binds to the PIF pocket of PDK1. Three molecules of valsartan produced a strong electron density at the PIF pocket and were modeled (Fig. 5J). Molecules #2 and #3 bind at locations occupied by PS48 and PS210 (fig. S9 A-D), compounds which did not affect PDK1 dimerization in our studies. Therefore, valsartan molecule #1, located on the helix α-B side of the PIF-pocket, could be responsible for disrupting PDK1 dimers (Fig. 5I,J). In agreement with this suggestion, Ala substitution of Val^124^, a residue in proximity of molecule #1, reduced the valsartan-induced thermal stabilization of PDK1 (fig. S9E, F).

### HYG8 stabilized a conformation of PDK1 with the PH domain sitting on the back of the catalytic domain

**Fig. 6:**
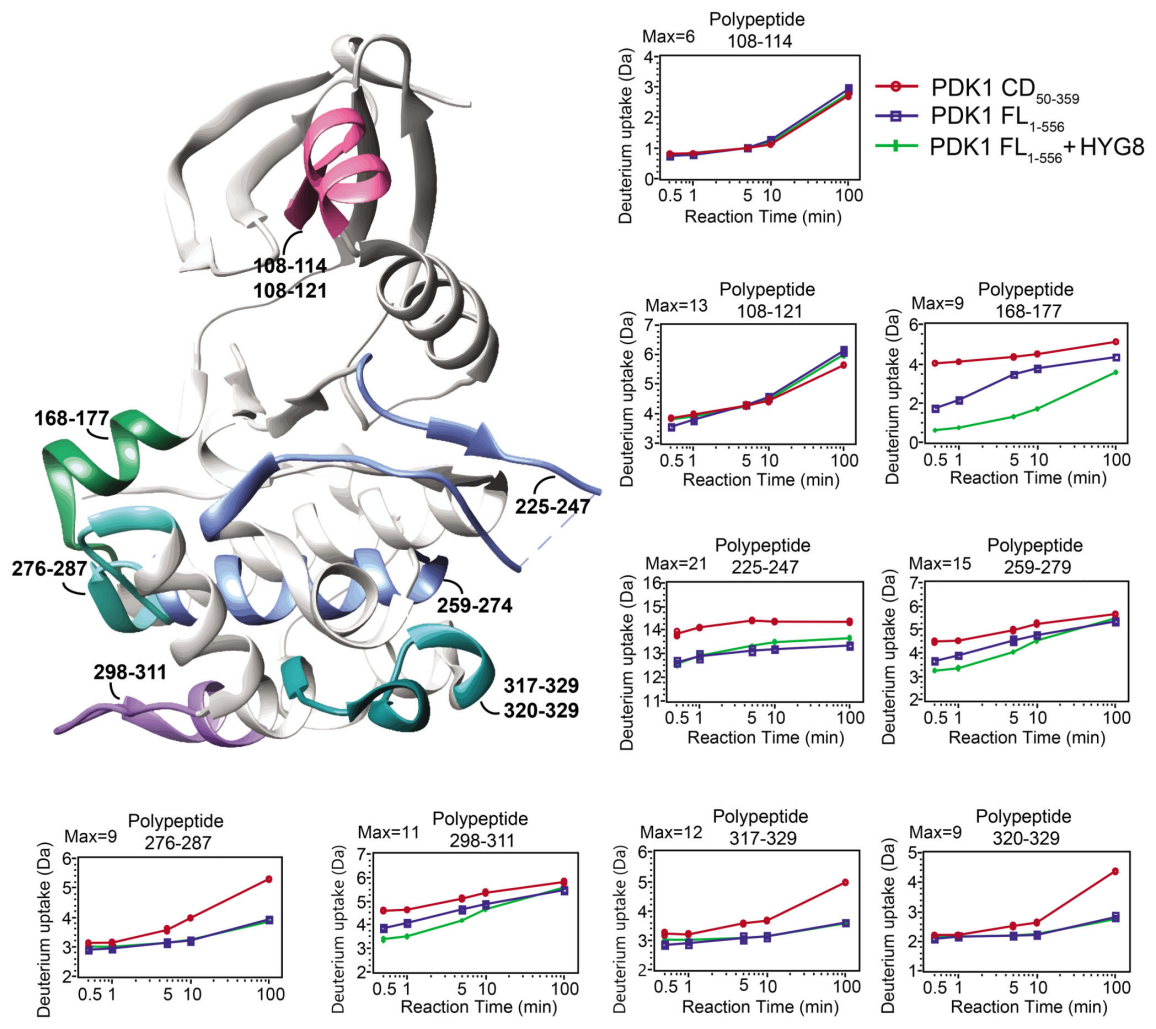
Hydrogen/deuterium (H/D) exchange identifies the site of interaction of the linker-PH domain with the catalytic domain that is stabilized by HYG8. H/D exchange was assessed for the catalytic domain of PDK1 (His-PDK1_50-359_), full-length PDK1 (His-PDK1_1-556_) and the full-length protein in the presence of HYG8 for 0.5, 1, 5, 10, and 100 min. The H/D exchange of selected polypeptides corresponding to the catalytic domain from PDK1_50-359_ (CD, red), PDK1_1-556_ (blue) and PDK1_1-556_ + HYG8 (green) are shown. The structure of the catalytic domain of PDK1 (PDB: 3HRC) is used to indicate the location of polypeptides. N=3 independent experiments.

We next used hydrogen/deuterium (H/D) exchange to investigate the conformation of full-length PDK1 in solution. We first compared the H/D exchange of the catalytic domain (PDK1_50-359_) to that of the catalytic domain polypeptides in full-length PDK1 (PDK1_1-556_). The H/D exchange of most polypeptides from the catalytic domain were not affected by the presence of the linker-PH domain in the PDK1_1-556_ construct. Notable exceptions were peptides 168-177, 225-247, 259-274 and 298-311, which were all more protected from H/D exchange in PDK1_1-556_ (Fig. 6, fig. S10A,B and fig. S11). Peptide 168-177, in which three hydrogens were protected from exchange, showed the highest degree of protection. The presence of HYG8 conferred protection to polypeptides 456-470 and 485-503, which correspond to the PIP_3_ binding site in the PH domain. HYG8 produced a further increase in the protection of peptide 168-177, 259-274 and 298-311. This result indicates that the linker-PH domain binds and protects from exchange polypeptides located at the back of the catalytic domain, whereas HYG8 binds to the PIP_3_-binding site of PH domain and stabilizes this conformation.

In the presence of HYG8, a small deprotection at the first time-point was observed on the peptide comprising residues 108-121, but not in the polypeptide comprising residues 108-114, indicating a deprotection in the region 114-121 which corresponds to the helix α-B. A similar H/D exchange in polypeptide 108-121 was observed in full-length PDK1 in the presence of HYG8 and on the corresponding peptide on the isolated catalytic domain. The protection at the back of the catalytic domain thus correlated with a deprotection of helix α-B, suggesting that in the presence of HYG8, the linker-PH domain could release a protection on helix α-B, a helix that constitutes the PIF pocket and that interacts with valsartan molecule #1. The above chemical biology studies indicate that PDK1 has at least three major conformations in equilibrium: monomers stabilized by HYG8, monomers stabilized by valsartan, and dimers. Moreover, these conformations are important because PDK1 conformations stabilized by HYG8-like compounds can selectively inhibit the phosphorylation of one substrate of PDK1, Akt. HYG8 is related to the headgroup of PIP_3_ (Fig. 2A). However, although PDK1 readily phosphorylates Akt in the presence of PIP_3_, HYG8 had the opposite effect. The head group of PIP_3_, inositol-1,3,4,5-tetrakisphosphate (IP_4_), did not enhance binding to PIFtide, indicating that IP_4_ did not stabilize the HYG8-bound conformation that inhibits the phosphorylation of Akt (fig. S12).

### The monomeric full-length PDK1 conformations stabilized by HYG8

**Fig. 7:**
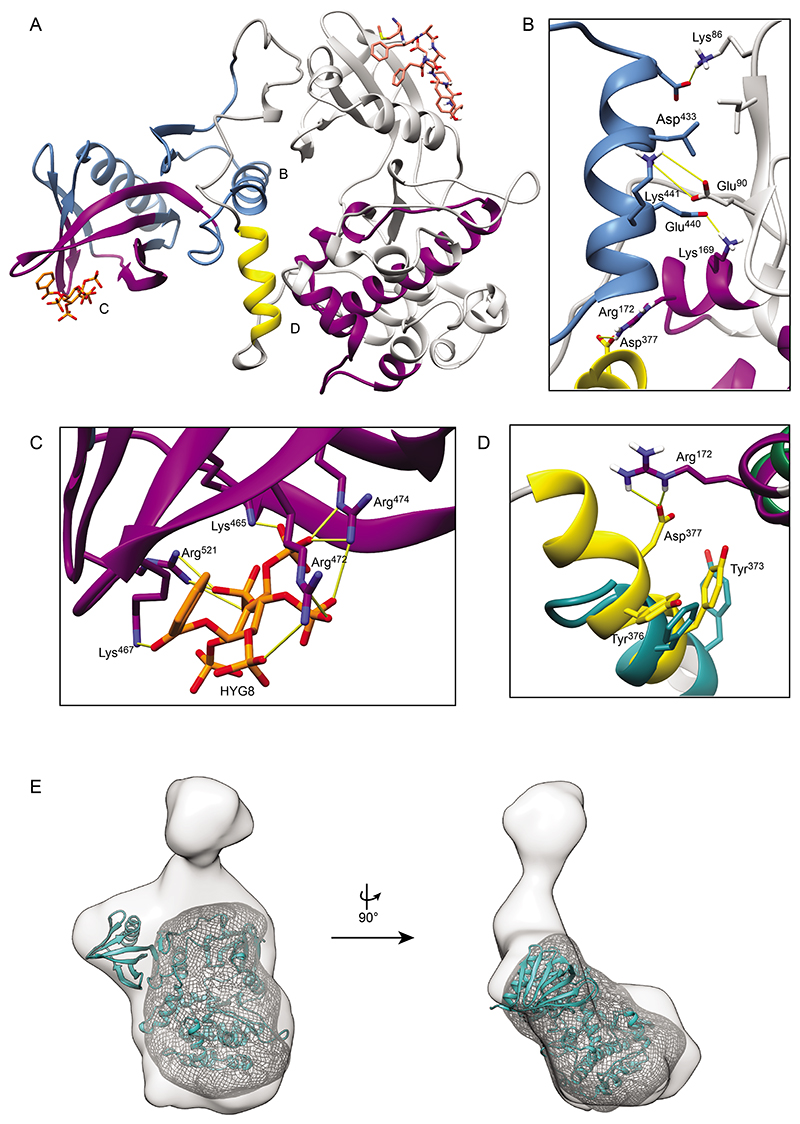
Molecular model of full-length PDK1 in complex with HYG8. (A) The modeled PDK1 structure (77-549) in the conformation bound to HYG8. The catalytic domain (dark grey) is modeled from PDB 4RRV and the PH domain (blue) is modeled from PDB 1W1G. The linker region (white cartoon) is a snapshot from an ensemble of conformations. Other colored regions are further explored with H/D exchange in fig. S11. (B) The interaction of the PH domain helix 434-443 (blue) with the catalytic domain. The conformation of the helix comprising (434-443) is from docking simulations using ClusPro. (C) HYG8 (orange, sticks) bound to the PH domain (docking). (D) The linker–catalytic domain interface was modeled based on the high affinity binding site of the pseudosubstrate inhibitor peptide PKI (5-24) from PKA (PDB 1CDK) (cyan) complexed to the catalytic domain of PKA (green). The PDK1 linker region (372-381) including Tyr^373^ and Tyr^376^ was modeled as a helix (yellow) occupying the equivalent hydrophobic pocket at the side of the catalytic domain that is occupied by PKI in PKA. (E) The modeled structure of PDK1 (residues 77-549) is shown fitted into the most representative ab initio low resolution structure DAMMIF (Fig. 4K) determined from experimental SAXS data for His-PDK1_1-556_ + HYG8 (light grey). The DAMMIF corresponding to the low resolution structure of the catalytic domain PDK1_50-359_ is shown in a grey mesh. The top part of the ab initio structure, not occupied by the model, is considered to be occupied by be the N-terminal 76 residues of PDK1 not present in the model and additional N-terminal residues from the His- and myc- tags present in the protein used for SAXS.

We modelled the structure of PDK1 (77-549) in the conformation stabilized by HYG8 (Fig. 7A). The structure comprises the catalytic domain, the linker region and the PH domain. The model was built based on a combination of available structural information obtained through crystallization, homology modeling, docking, and molecular dynamics simulations. The PH domain was specifically modeled from PDB ID 1W1G, in which it is bound to IP_4_ (39), and the catalytic domain was modeled from PDB ID 4RRV, in which it is bound to PIFtide and ATP (31). The model explains the protection of the polypeptide 168-177 and all experimental H/D exchange data (Fig. 6 and fig. S11).

In our model, the binding site on the PH domain for PIP_3_, inositol phosphates and the HYG compounds is distant from the region that interacts with the catalytic domain. Thus, we predict that the binding of HYG8 allosterically affects the helix 434-443 to enhance the intramolecular interaction of the PH domain with the catalytic domain of PDK1 (Fig. 7B,C). This helix is unique to the PH domain of PDK1 (39). Part of the linker region including Tyr^373^ and Tyr^376^ (^372^CYGNYDNLLS^381^) was modeled to attach to the catalytic domain in a manner analogous to the Tyr^7^ and Phe^10^ residues that provide a high affinity binding to the pseudosubstrate inhibitor peptide PKI (5-24; ^5^TTYADFIASGRTGRR^24^) that binds to a homologous kinase cAMP-dependent protein kinase, PKA (PDB ID 1CDK) (57) (Fig. 7D). The remainder of the linker was modeled using docking and implicit solvent simulations. Other than the helix at 372-381, the linker region is flexible and better represented as an ensemble of structures. Tyr^373^ and Tyr^376^ correspond to phosphorylation sites on PDK1, and the depicted conformation of PDK1 with the 372-381 segment selected for the model can only be achieved when both Tyr residues are not phosphorylated.

The general orientation of the catalytic domain relative to the linker-PH domain in the AlphaFold (58) model of full-length PDK1 was overall similar to our modeled structure (fig. S8F). Furthermore, the AlphaFold model also explained the protection from H/D exchange of the peptides 168-177, 259-274 and 298-311 (Fig. 6). AlphaFold also models an interaction of the linker-PH domain with the pocket on the catalytic domain that we modeled to interact with Tyr^373^ and Tyr^376^. This pocket was also occupied in the AlphaFold models of full-length PDK1, even in distant orthologs that have a zinc-finger domain instead of a PH domain. In the AlphaFold model, however, Tyr^373^ and Tyr^376^ were relatively exposed, suggesting that the AlphaFold conformation could explain the structure of a form phosphorylated at the Tyr residues. Both modeled conformations were active overall and the peptide substrate binding site was exposed for peptide substrate binding, in agreement with HYG8 not inhibiting the catalytic activity of PDK1. The modeled conformations could also bind the HM of substrates of PDK1 as observed in the crystal structures (31, 32) and fit with the biochemical information indicating that HYG8 and PIFtide could bind to the same conformation. They were also consistent with the finding that PDK1 in complex with HYG8 could efficiently phosphorylate SGK, which requires a docking interaction of its HM with the PIF pocket of PDK1. On the other hand, even if the enzyme is active, the biochemical data indicated that the conformation stabilized by HYG8 did not phosphorylate Akt in the absence of PIP_3_, suggesting that this conformation, with the linker-PH domain associated to the back of the catalytic domain, hinders the specific interaction with Akt but not the interaction with SGK. Our modeled structure (PDK1_77-549_) could be fitted into the low-resolution structure of full-length PDK1_1-556_ from SAXS in the presence of HYG8 obtained above, with the large lobe of the catalytic domain occupying the more rounded bottom of the structure (Fig. 7E). The fitting of the ribbon model and the simulated dynamics to the envelope do not appear perfect (Fig. 7E and fig. S8H). This could be due to imperfections of the model, which is possible due to the number of assumptions that were used for building the model. Alternatively, the discrepancies may be due to the actual envelope, which represents the average of dynamic regions in conformations stabilized by HYG8. Since the allosteric nature between the PIF-pocket and the ATP-binding site is conserved, we must expect that the envelope may represent an average between open and closed conformations. In addition, we note that the regions that seem to extrude from the envelope, correspond to regions of the protein that are very dynamic, as resolved in previous HDX experiments (54) and in MD simulations (32) (fig. S8G).

## Discussion

Protein kinases are often multidomain proteins, in which the different domains play roles in the regulation of activity. However, in most cases the mechanisms of regulation are poorly understood, despite their importance to signaling and disease. The PH domain of PDK1 is required for co-localization of PDK1 with one of its substrates, Akt, and for PDK1 dimerization (22, 45, 46, 48). Our present study showed that full-length PDK1 could adopt at least three conformations, which were determined by the position of the linker and the PH domain. The monomeric conformations of PDK1 were biologically relevant because they had distinct biochemical properties. The HYG8-stabilized conformation bound to PIFtide and was active towards SGK but not Akt, whereas the second monomeric conformation did not bind PIFtide and therefore could not interact with and phosphorylate substrates that require the docking interaction, such as S6K, SGK, RSK and PKC isoforms. Together, the characteristics of the two monomeric conformations of PDK1 reveal a new mechanism of substrate-selective regulation, mediated by the position of the linker PH domain on the catalytic domain. The various conformations provide an additional mechanism to adjust the selective and timely phosphorylation of PDK1 substrates. Here, we depicted the molecular structure of one of the PDK1 monomeric forms in which the PH domain associates with the back of the catalytic domain. Together, these biochemical and structural findings can also be harnessed for the rational development of substrate selective drugs. Previously characterized compounds binding to the PIF pocket of PDK1 are substrate-selective inhibitors that block the phosphorylation and activation of substrates that require docking to the PIF pocket. In contrast, compounds stabilizing PDK1 in the conformation modeled in complex with HYG8 are predicted to have the opposite effect of selectivity, inhibiting Akt phosphorylation without affecting the phosphorylation of other substrates of PDK1.

The finding that HYG8 “enhanced” binding to PIFtide several fold indicated to us that, in the absence of added compounds, only a minor proportion of PDK1 was in a conformation suitable for binding to PIFtide and that the addition of HYG8 enriched PDK1 in conformations that were competent for binding to PIFtide. Although we deduced the existence of two monomeric conformations of PDK1, we only modeled the conformation of PDK1 in the presence of HYG8. Unfortunately, we could not obtain reliable H/D exchange data in the presence of valsartan. However, we can deduce some characteristics of the second monomeric conformation of full-length PDK1. The SEC-SAXS of full-length PDK1 in the absence of HYG8 shows that the apo structure is similarly compacted to the HYG8-bound conformation, excluding the possibility that the linker-PH domain region is randomly flexible and indicating that the linker-PH domain must also fold onto the catalytic domain in the second monomeric conformation of PDK1. In addition, three sets of data suggest that the second monomeric conformation of PDK1 may have the linker-PH domain region attached to the small lobe of the kinase, in the proximity of the PIF-pocket. First, valsartan bound at the PIF pocket and stabilized full-length PDK1 to a greater extent than the catalytic domain, suggesting that valsartan could interact with both the PIF pocket and the linker-PH domain. Second, in the presence of HYG8, concomitant with protection at the back of the kinase, there was a deprotection at the helix α-B. We can speculate that by attaching to helix α-B, the linker-PH domain could block the binding of PIFtide and still bind PS48 and PS210. In such a model, valsartan molecule #1, which interacted with the helix α-B but also extended outwards, could act as a glue between the catalytic domain and the linker-PH domain to stabilize this conformation. Third, most of the conformations proposed by the EOM evaluation of SEC-SAXS data of apo-PDK1 localized the linker-PH domain interacting with the small lobe of the kinase.

Levina *et al*. used a construct fusing PDK1 to PIFtide to stabilize PDK1 dimers with the aim of investigating the transient PDK1 dimer that must form during in vitro trans-autophosphorylation of the activation loop (52). In the proposed model by Levina *et al*., a putative hydrophobic motif sequence from the linker region binds to the PIF pocket of the neighbouring PDK1 molecule. In this situation, PIFtide binding with high affinity to the PIF pocket should displace those dimers. The model proposed by Levina *et al*. is not supported by our data because PIFtide did not disrupt dimers. Also, the Levina *et al*. model predicts that the addition of PIFtide or an equivalent HM polypeptide would inhibit PDK1 autophosphorylation. However, Frödin *et al*. showed that a HM polypeptide enhances PDK1 autophosphorylation (59). Therefore, the model described by Levina *et al*. does not fit the characteristics of the PDK1 dimers presented by this study or previously published biochemical studies. In the present work, we investigated PDK1 dimers using AlphaScreen technology. In addition, we also validated the presence of PDK1 dimers and the disruption of dimers by HYG8 by using AlphaScreen assays, batch SAXS and a super resolution microscopy STORM-based method. Thus, we are confident that the AlphaScreen interaction assays between PDK1 constructs shown in this work represent PDK1 dimers that are disrupted by HYG8.

Levina *et al*. also proposed that the PH domain autoinhibits PDK1 (52), which contrasts with previous studies showing that constructs lacking the PH domain phosphorylate diverse substrates to a similar extent in vitro, including peptide substrates, such as T308tide (28, 51). Given the results presented here, autoinhibition based on the decreased phosphorylation of one substrate may not reflect the inhibition of the catalytic activity but the contribution of a conformation of full-length PDK1 to the phosphorylation of one particular substrate or a subset of substrates.

Here, we also observed that the phosphorylation of peptide substrates was affected by the PDK1 construct (fig. S1, A-E) and that the binding of HYG8 disrupted the interaction of a particular peptide substrate with PDK1_1-556_ (fig. S1, F,G), indicating that the interaction is affected by the conformation of full-length PDK1. Similar to the polypeptides GS-022 and GS-023, we suggest that the commercial polypeptide substrate Ser/Thr 07 may be an excellent substrate due to the combination of a low affinity interaction at the peptide substrate binding site and a second important interaction with the linker-PH domain region of PDK1 at epitopes that are hidden in the conformations stabilized by HYG8. In this manner, binding of Ser/Thr 07 to PDK1 is conformation-dependent, such that interaction with PDK1 and the rate of phosphorylation in the presence of HYG8 is decreased.

In our model, the binding of HYG8 to the PH domain induced an allosteric effect, enhancing the interaction of the linker-PH domain with the back of the catalytic domain. By stabilizing this monomeric conformation, HYG8 induced the observed enhancement of binding to PIFtide. In contrast, we concluded that the binding of the headgroup of PIP_3_, IP_4_, did not produce the same conformational change (fig. S12). Thus, the second monomeric conformation was compatible with binding to PIP_3_ at the cell membrane and could be responsible for Akt phosphorylation. Finally, the phosphorylation of other substrates of PDK1 that require the docking interaction (such as SGK, S6K, RSK and PKC isoforms) could be catalyzed by the HYG8-stabilized conformation or by PDK1 dimers but not by the second monomeric conformation.

Compounds that bind to the PIF pocket, such as PS210 and RS1, are substrate-selective inhibitors, inhibiting the phosphorylation of substrates such as SGK and S6K, but not that of Akt (29, 31). Based on our in vitro data, we suggest that compounds that stabilize PDK1 in a similar way to HYG8, or alternatively, compounds that directly stabilize the PH domain-catalytic domain interface, would have the opposite effect on selectivity as compounds that bind to the PIF pocket - inhibiting Akt signaling without affecting the phosphorylation of SGK. In cells, the conformation of PDK1 that does not bind PIFtide could still phosphorylate Akt but would be impaired in the phosphorylation of substrates that require a docking interaction with the PIF pocket. Although not as potent as HYG8 and HYG14, the cellular metabolite IP_5_ displaced PIP_3_ and disrupted PDK1 dimers in vitro. It is tempting to speculate that IP_5_ could also selectively inhibit Akt phosphorylation by PDK1 under certain cellular situations in which IP_5_ levels reach local concentrations of 50-100 nM (39). We can also speculate that the PDK1 conformations here described may be stabilized by post-translational modifications, interactions with metabolites or interactions with additional proteins, to fine-tune PDK1 cellular signaling. Future work should uncover the location, mechanism of regulation and how these conformations are biologically regulated. Regardless, we predict that stabilizing PDK1 monomeric conformations with drugs that act like HYG8 or valsartan could lead to selective inhibition of PDK1 activity, differentially affecting the phosphorylation of different substrates. More potent and cell-permeable compounds will be required to investigate the pharmacological effects of compounds stabilizing these monomeric conformations of PDK1.

Cellular studies have been performed on two PDK1 mutants with alterations within the PH domain: T513E (47, 48, 50, 51), which is mutated at a predicted phosphorylation site, and K365E, which involves a charge reversal on a Lys residue important for the interaction with the phosphates of PIP_3_ (23, 60, 61). In vitro, neither mutation affects the specific activity of PDK1 when measured using T308tide as a substrate (23, 51). Notably, the K465E mutant shows increased binding to PIFtide (62). In cells, the T513E mutant has increased basal activity towards Akt, without increased membrane localization (50) whereas the K465E mutant shows residual phosphorylation activity towards Akt, which is independent of the binding to PIP_3_ (63). Considering our new findings, we suggest that the increased phosphorylation of Akt by these PDK1 mutants could be mediated by an alteration in the equilibrium between PDK1 conformations, increasing the proportion of conformations of PDK1 that are suitable for phosphorylation of Akt by a mechanism involving PIF-pocket docking interactions.

**Fig. 8:**
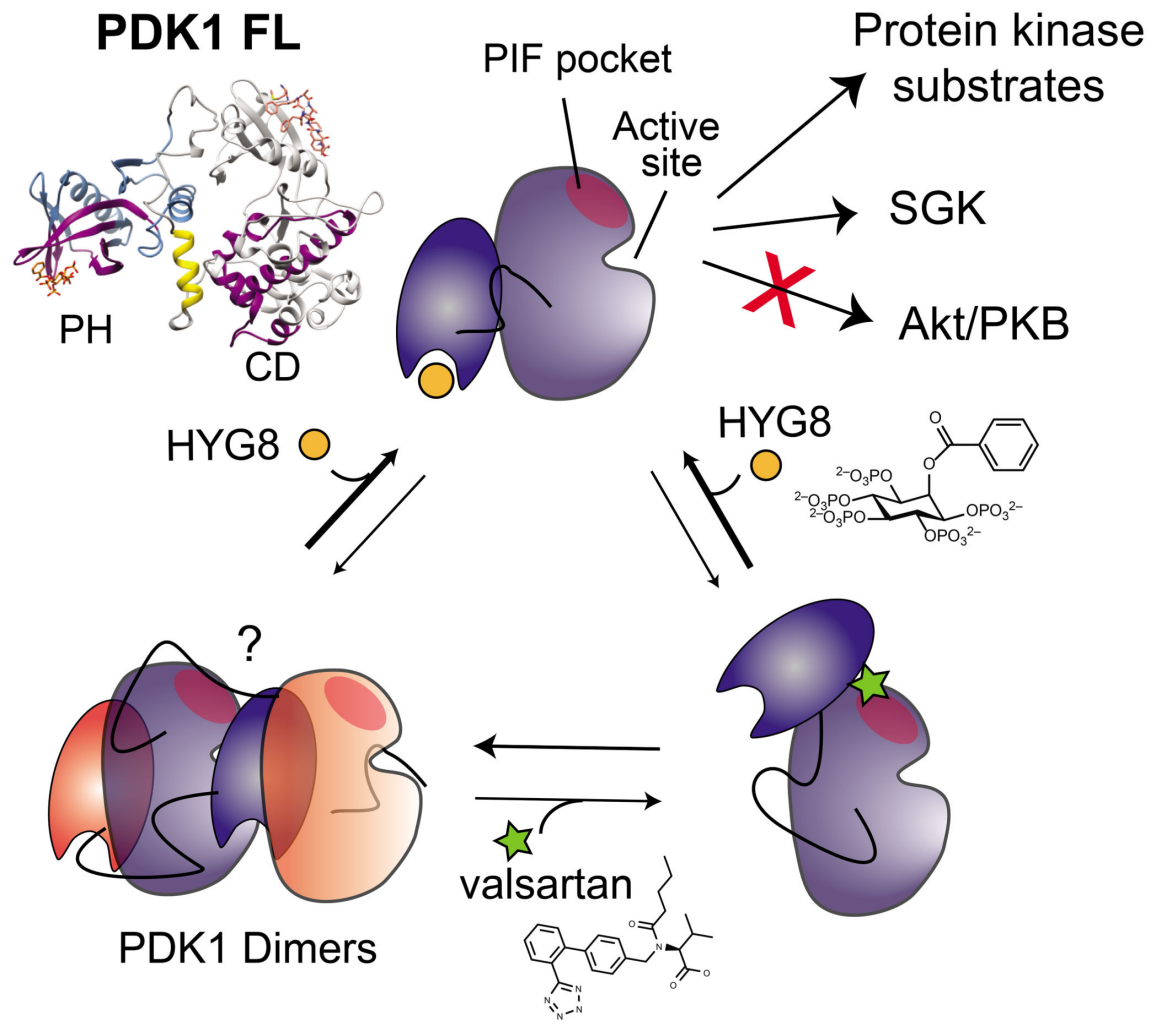
Different conformations of full-length PDK1 determine substrate specificity. Small molecules HYG8 and valsartan stabilized different monomeric conformations of full-length PDK1, defined by the relative position of the linker-PH domain. The different conformations of full-length PDK1 had distinct biochemical characteristics. The conformations of PDK1 stabilized by HYG8 protected the “back” of the kinase catalytic domain, had unmodified in vitro activity towards some substrates, such as SGK, but were impaired in the phosphorylation of Akt (also termed PKB) and another particular peptide substrate.

As an important member of the PI3K signaling pathway, PDK1 has been considered a target for anti-cancer drug development. However, PDK1 inhibitors have not advanced in clinical trials. Because PDK1 is necessary for the activity of many kinases that take part in diverse signaling pathways, complete inhibition of PDK1 may have substantial on-target, unwanted side-effects. Here, we uncovered the possibility of stabilizing particular conformations of PDK1 monomers, which would have differential effects on substrates with distinct mechanisms of phosphorylation by PDK1 (Fig. 8). This work thus identifies strategies to inhibit PDK1 downstream signals selectively with small molecules and more broadly, to develop more selective drugs directed against protein kinases. This knowledge could be used to develop innovative drugs for combination treatment of patients with a broad range of cancers.

## Materials And Methods

### Materials

The polypeptide substrate used for PDK1 was T308tide (KTFCGTPEYLAPEVRR; > 75% purity). The peptide substrate used for SGK and Akt was KK-Crosstide (KKGRPRTSSFAEG). Other polypeptides used were PIFtide (REPRILSEEEQEMFRDFDYIADWS), a peptide derived from the PRK2 HM (a PDK1 substrate), and substrates GS-022 (RRRQFSLRRKAK), and GS-023 (RRRQFSLRRKA-K(5-FAM). Peptides used in AlphaScreen interaction assays were biotin-PIFtide (biotin- REPRILSEEEQEMFRDFDYIADWS) and biotin-GS-022 (biotin-RRRQFSLRRKAK). Peptides were synthesized by Pepscan. PS210 was synthesized by us and characterized previously (29, 30) and PS653 was from Maybridge (32). Biotin-C6-PIP_3_ was from Echelon Biosciences. IP_5_ (64), *scyllo*-IP_5_ (64), 2-*O*-(2-aminoethyl)-IP_5_ (AMR1474) (43), 2-*O*-butyryl-IP_5_ (HYG7) (65) and 2-*O*-benzoyl-IP_5_ (HYG8) (66) were synthesized and characterized as previously reported. IP_6_ was obtained commercially (Sigma) and further purified by gradient elution from a Q-Sepharose Fast-Flow resin and isolated as the triethylammonium salt before use. The syntheses and characterization of 2-*O*-acetyl-IP_5_ (HYG6) and 2-*O*-butyl-IP_5_ (HYG14) are described in the Supplementary Materials. All inositol phosphates and derivatives were fully characterized by ^1^H, ^13^C and ^31^P NMR spectroscopy and used as their triethylammonium salts. Antibodies were purchased from Cell Signaling (#13038, #5642) and secondary antibodies from LI-COR (926-68022 and 926-32213). AlphaScreen beads were purchased from Perkin Elmer (6760619, 6765301, 6760106). SYPRO-Orange 5000x was purchased from Invitrogen™ (S6650). Valsartan was purchased from Sigma (SML0142) and des(oxopentyl)valsartan from Carbosynth. Complete protease inhibitor cocktail tablets were from Roche. Protein concentration was determined using Bio-Rad Protein Assay Dye Reagent Concentrate (#50000069). Ni-NTA was from Jena Bioscience and glutathione sepharose resin was from GE Healthcare. SNAP-Cell® TMR-Star was purchased from NEB (#S9105). Human embryonic kidney (HEK) 293 cells (ATCC) were cultured in Dulbecco’s modified Eagle’s medium containing 10% fetal bovine serum and PenStrep. Mammalian tissue culture materials were from Greiner and JETBiofil. Insect cell expression system and all the insect cell related material were from Invitrogen (Thermo Fisher Scientific). DNA constructs used for transient transfection were purified from bacteria using a Qiagen plasmid Maxi kit.

### General Chemistry Methods

Reagents and solvents were of either commercial quality obtained from Sigma-Aldrich (Gillingham, Dorset, U.K.) or Acros-Fisher Scientific (Loughborough, U.K.). Petroleum ether (40-60 °C) is abbreviated as pet. ether. NMR spectra were recorded with a JEOL EX-270 or a Varian Mercury VX 400 or Bruker Avance III (400 MHz and 500 MHz) spectrometer. ^1^H NMR and ^13^C NMR chemical shifts are measured in ppm (δ) relative to internal tetramethylsilane (TMS) and ^31^P NMR chemical shifts are measured in ppm (δ) relative to phosphoric acid as an external standard. Signals are expressed and abbreviated as s (singlet), d (doublet), t (triplet), q (quartet), m (multiplet), br (broad) and ap (apparent). All ^1^H NMR and ^13^C NMR assignments are based on COSY, HMQC, HMBC and DEPT experiments. Coupling constants (J) are given in Hz. HRMS mass spectra were recorded on a Bruker MicroTOF spectrometer. Melting points (m.p.) were determined using a Reichert-Jung hot stage microscope apparatus or a Stanford Research Systems Optimelt MPA100 automated melting point system and are uncorrected. Thin-layer chromatography (TLC) was performed on pre-coated plates (Merck TLC aluminium sheets, silica gel 60 F254) with detection by UV light or with phosphomolybdic acid in ethanol followed by heating. Flash chromatography was performed on silica gel (particle size 40-63 μm) using glass columns or on an ISCO CombiFlash Rf automated flash chromatography system using RediSep Rf disposable flash columns. Ion-exchange chromatography was performed on a BioLogic LP low-pressure chromatography system, eluting with gradients of triethylammonium bicarbonate (TEAB) buffer and using Milli-Q quality water. 2 M TEAB (pH 7.8) was prepared by bubbling carbon dioxide gas into 2 M triethylamine solution. Water used in the purification of water-soluble polyphosphates was of Milli-Q quality. Phosphate-containing fractions were identified using a modification of the Briggs phosphate test (67) and the target polyphosphates were accurately quantified using the Ames phosphate assay (68).

### Expression and purification of protein kinases

His-PDK1_1-556_ employed in kinase activity assays and in the AlphaScreen interaction assays was expressed in Sf9 insect cells using the Bac-to-Bac™ baculovirus expression system (Invitrogen/Thermo Fisher) and purified through Ni-NTA and gel filtration chromatography, as previously described (28, 29). His-PDK150-556 fused to SNAP-tag was expressed in insect cells and purified as described for His-PDK1_1-556_. GST-PDK1_360-556_ (GST-PDK1-Linker-PH) was cloned into the BamHI-KpnI sites in pEBG2T plasmid. The GST-fusion proteins (GST-PDK1_1-556_, GST-PDK1_360-556_, GST-SGK [S422D]; GST-Akt/PKB [S473D] and GST-Akt/PKB ΔPH[S473D]) were obtained from HEK293 cells after transient transfection of the corresponding pEBG2T plasmids and purified as previously described (28). Cells expressing the last 3 constructs were serum starved 24 h before cell lysis to lower phosphorylation of the active site. GST-PDK1_1-556_ and GST-PDK1_360-556_ used in AlphaScreen assays were dialyzed to remove the remaining GSH after purification. Bacterially expressed PDK1 PH domain (residues 408-556) used in fig. S3A was expressed as a GST-fusion protein from pGEX-6P1 (plasmid provided by Dundee University through MRC UK to B.V.L.P.) and the GST-tag cleaved with PreScision protease as previously described (69). Purified proteins were aliquoted, snap frozen in liquid nitrogen and stored at -80 °C until use.

### In vitro PDK1 activity tests

The PDK1 activity assay using T308tide as a substrate was performed essentially as previously described (18, 28, 54). In brief, the assay was performed in a 20 μl format containing 50 mM Tris pH 7.5, 0.05 mg/ml BSA, 0.1% ß-mercaptoethanol, 10 mM MgCl_2_, 100 μM [γ^32^P]ATP (5-50 cpm/pmol), 0.003% Brij, 150-500 ng PDK1, and T308tide (0.3 mM) at room temperature. The assay was performed in 96 well plated and stopped by the addition of 5 μl of 0.1 % phosphoric acid. 4 μl aliquots were spotted on p81 phosphocellulose papers (Whatmann) using ep motion 5070 (Eppendorf). The phosphocellulose papers were washed in 0.01% phosphoric acid, dried, and exposed using PhosphoImager (FLA-9000 Starion). Data were analyzed with ImageQuant software (Fujifilm). Activity measurements were performed in technical duplicates or triplicates with less than 10% difference between replicates. Experiments were repeated at least twice. The activity measurements of PDK1 towards GS-022 and GS-023 were performed similarly, with the GS-peptides replacing T308tide at the indicated concentrations. GS-022 and GS-023 have features consistent with the loose characteristics disclosed for the peptide Ser/Thr 07, ((R/K)1-3(X)0-2 (S/T) (X)0-2(R/K)1-3, where X is any residue) used by the commercial “PDK1 Direct” assay.

PDK1 activity was also assessed in a cascade format that measures the ability of PDK1 to activate its substrates GST-ΔN-SGK [S422D], GST-Akt [S473D] and GST-Akt ΔPH [S473D], as previously performed (24, 28). SGK and Akt proteins mutated at the HM phosphorylation site to Asp can be activated by phosphorylation at the activation loop by PDK1. In brief, we preincubated SGK or Akt (0.5 μg) with 10 ng or 100 ng of PDK1 at room temperature (22°C) in a buffer containing 50 mM Tris pH 7.5, 0.05 mg/ml BSA, 0.1% β-mercaptoethanol, 10 mM MgCl_2_, 100 μM ATP (20 μl). We measured the activity of SGK or Akt by adding 30 μl buffer containing the polypeptide substrate KK-Crosstide (KKGRPRTSSFAEG; 100 μM) and [γ^32^P]ATP (100 μM). The activity assay was stopped, spotted on p81 phosphocellulose papers and processed as above for the assay using T308tide. As a control, SGK and Akt were preincubated in the absence of PDK1. The activation of SGK and Akt is the difference between the basal activity and the activity of the substrate kinases after PDK1 phosphorylation.

The commercial PDK1 activity assays were provided by Thermo Fisher Scientific as a service. In the cascade assay format full-length His-PDK1 phosphorylates inactive full-length Akt2, which in turn phosphorylates a synthetic peptide substrate (Ser/Thr 06). The assay is performed in the absence of lipid vesicles in a buffer containing 5-20 ng PDK1, 150 ng inactive Akt2, and 2 μM Ser/Thr 06 in 50 mM HEPES pH 7.5, 100 μM ATP, 0.01% Brij-35, 10 mM MgCl_2_, 1 mM EGTA. The ¨PDK1 Direct¨ biochemical assay measures the phosphorylation of a peptide termed Ser/Thr 07 that is labeled with a donor fluorophore (coumarin) and an acceptor fluorophore (fluorescein) to create a FRET pair. The peptide substrate was first incubated with the kinase and ATP-Mg (25 μM ATP), then non-phosphorylated peptide was cleaved with a protease. The remaining FRET corresponds to the phosphorylated substrate. The information from the company states: “The final 10 μL Kinase Reaction consists of 5.04 - 29.4 ng PDK1 Direct and 2 μM Ser/Thr 07 in 50 mM Tris / HEPES pH 8.0, 0.01% BRIJ-35, 10 mM MgCl_2_, 1 mM EGTA, 0.01% NaN_3_. After the 1 h Kinase Reaction incubation, 5 μL of a 1:65000 dilution of Development Reagent A is added.” Each compound concentration was tested twice. A control standard curve (10 point titration) of the inhibitor staurosporine was run on the same plate to ensure that PDK1 was inhibited within the expected range. IC_50_ of staurosporine was 8.89 nM. Additional controls that were included on the same plate are: 0% Phosphorylation, 100% Phosphorylation, 0% Inhibition and control tests for interference with the development reaction and fluorescence detection. Thermo Fisher Scientific does not provide the identity of Ser/Thr 07.

PDK1 activity was also measured by incubating PDK1 with protein kinase substrates and measuring their phosphorylation by Western blot using phospho-specific antibodies to the activation loop phosphorylation site, essentially as previously performed (24), with the modification that the secondary antibodies were fluorescent for improved quantification. In brief, we incubated the purified protein substrates of PDK1 with GST-PDK1_1-556_ in the presence or absence of HYG8 (0-1 μM) for 10 min at 30°C in a buffer containing 50 mM Tris-Cl pH 7,4; 50 mM MgCl_2_; 100 μM ATP; 0.1 % v/v 2-mercaptoethanol. The conditions of PDK1 phosphorylation of substrates were chosen to be in the linear range. GST-Akt S473D (125 nM) and GST-Akt S473D ΔPH (125 nM) were phosphorylated by 1 nM GST PDK1, whereas GST-SGK S422D (1 μM) was phosphorylated by 0.05 nM GST PDK1. The reaction was stopped by the addition of Laemmli SDS-PAGE sample buffer and boiling the samples for 5 min at 95 °C. The basal phosphorylation of substrates was determined in the absence of PDK1, while “maximal” phosphorylation corresponds to the phosphorylation of substrates with excess PDK1 (10 nM PDK1 for Akt/PKB substrates and 1 nM PDK1 for SGK). Samples (300 ng) were run on 10% acrylamide gels, separated by SDS-PAGE, and transferred to nitrocellulose membranes. Membranes were incubated with primary antibodies [phospho-Akt (Thr^308^) rabbit monoclonal antibody (#13038 Cell Signaling) and phospho-SGK3 (Thr^320^) rabbit mAb (#5642 Cell Signaling)] overnight at 4 °C, then with secondary antibody (800CW donkey anti-rabbit from Licor). Fluorescent signals were measured using Odyssey Fc (Licor). Image Studio software was used to quantify band intensities. The percentage of phosphorylation was normalized to the 0 nM HYG8 condition.

### Lipid Overlay Assays

Dots containing 2-1000 picomoles of di-C_18_-PIP_3_ (70) were prepared by spotting 1 μL of serial dilution stock solutions (2-1000 μM dissolved in a 1:2:0.8 by volume mixture of chloroform/methanol/water) onto Hybond-C extra membrane (GE Healthcare), which were dried at room temperature. Membranes were incubated with blocking buffer (50 mm Tris-HCl buffer pH 7.5, 2 mg/ml fatty acid free bovine serum albumin, 150 mM NaCl, and 0.1% (v/v) Tween 20) for 1 h at room temperature. Membranes were then incubated with 10 nM protein/10 μM inhibitor mixes in blocking buffer with gentle rocking overnight at 4 °C. Membranes were washed 10 times over 1 h at room temperature using TBST buffer (50 mM Tris-HCl pH 7.5, 150 mM NaCl, 0.1% (v/v) Tween 20) before being incubated for 1 h with 1:2000 dilution of rabbit recombinant anti-PDK1 monoclonal antibody (New England Biolabs). Membranes were washed and incubated with 1:2000 dilution of anti-rabbit HRP conjugated antibody (Invitrogen/Thermo Fisher) for 1 h at room temperature. Finally, after washing, proteins bound to the membranes were detected using Novex ECL chemiluminescent reagent (Invitrogen/Thermo Fisher) and developed on X-ray film.

### PDK1 interaction-displacement assays using AlphaScreen technology

The AlphaScreen® assay was performed following the general guidelines from the manufacturer (Perkin Elmer) (29). In short, the assay was performed in a final volume of 25 μl in white 384-well microtiter plates (Greiner) in a standard buffer containing 50 mM Tris-HCl (pH 7.4), 100 mM NaCl, 2 mM DTT, 0.01% (v/v) Tween-20 and 0.1% (w/v) BSA, with the addition of 5 μl of beads (anti-GST coated acceptor beads and streptavidin-coated donor beads; nickel chelate-coated acceptor beads and streptavidin-coated donor beads; or nickel chelate-coated acceptor beads and GSH-coated donor beads, 20 μg/ml final concentrations for each bead type). AlphaScreen assays for His-tagged proteins and GST-fusion proteins were performed using nickel chelate-coated acceptor beads and GSH-coated donor beads; alphascreen assays for His-tagged proteins and biotinylated molecules were performed using nickel chelate-coated acceptor beads and streptavidin-coated donor beads; alphascreen assays for GST-fusion proteins and biotinylated molecules were performed using anti-GST coated acceptor beads and streptavidin-coated donor beads (Perkin Elmer).

The conditions for each AlphaScreen assay were identified in cross-titration experiments for each interaction to choose the ideal pair of concentrations within the linear range of the interactions. As required, the standard conditions were modified to use less material, to increase binding of the interacting molecules, to lower background signal, and so on. His-PDK1 and GST-fusion protein in the presence or absence of the different compounds were incubated in the dark for 60 min at room temperature and the emission of light from the acceptor beads was measured in an EnVision reader (Perkin Elmer) or Spark plate reader (Tecan). AlphaScreen assays were performed at least twice, although most experiments were performed multiple times by different researchers with different batches of purified proteins with similar results.

Biotin-PIP_3_ interactions with GST-PDK1_1-556_ or GST-PDK1_360-556_ were assessed in the standard buffer. For the interaction between His-PDK1_1-556_ and GST-PDK1_1-556_, we explored variations on assay conditions that would stabilize the dimer/oligomer interactions of PDK1. We saw a good interaction at pH 7.4 with 150 mM NaCl, 0.1% (w/v) BSA, 0.05% (v/v) Tween20, and 2 mM DTT.

The screening of the Prestwick Chemical Library, a library of 1520 mainly FDA-approved drugs, was performed using the His-PDK1_1-556_ and GST-PDK1_360-556_ interaction assay in the standard buffer. Compounds were tested at 50 μM in a final concentration of 1% DMSO in all wells. Compounds were tested in a single replicate and positive hits were retested in duplicates to confirm results. Positive hits were compared against previous screening assays and repeated hits were discarded for being regarded as unspecific. Hit compounds were chosen for purchasing based on potency and chemical variability. The compounds that were purchased were tested in the assay to confirm the result.

The interaction between GST-PDK1_1-556_ and biotin-PIFtide was performed as described previously (29, 32) in the standard buffer, but the bead concentration was lowered to 15 μg/ml. Interaction of GST-PDK1_1-556_ and GST-PDK1_1-359_ (both at 150 nM) with biotin-GS-022 (at the indicated concentrations) was performed in the standard buffer and at bead concentrations suggested by Perkin Elmer.

### Differential scanning fluorimetry (DSF)

The thermal stability of PDK1 was assessed by monitoring the unfolding of PDK1 by the increase in the fluorescence of SYPRO Orange (Invitrogen) using a real-time PCR device (StepOnePlus, Applied Biosystems and BioRad CFX96) following a previously described protocol (70). The reactions were performed in a final volume of 10 μl in white 96-well PCR microtiter plates (Greiner) and contained 1 μM PDK1, 10 mM HEPES (pH 7.5), 150 mM NaCl, 1/1000 SYPRO Orange and 1 mM dithiothreitol. For valsartan, the assay buffer was modified to exclude NaCl. PIFtide or compounds (at a final DMSO concentration of 1%) were added to this reaction mixture. The temperature gradient was performed in steps of 0.3 °C in the range from 25 to 70 °C. To calculate the T_m_ values, data were exported to GraphPad Prism and curves fitted to a Boltzmann sigmoidal equation with an acceptable confidence interval of 95%. Statistical analysis was performed with Infostat software (http://www.infostat.com.ar) and GraphPad Prism by first verifying a normal distribution of pooled residuals (Shapiro-Wilks test) and homogeneity of variance (F test), and then performing a one-way ANOVA followed by the post hoc comparisons test indicated in each figure legend.

### X-ray crystallography

Crystallography of PDK1_50-359_ and soaking experiments with valsartan were performed essentially as previously described (32). PDK1_50-359_ used for crystallography was expressed in insect cells and purified through Ni-NTA affinity chromatography, followed by dialysis in 50 mM Tris-HCl pH 7.4, 500 mM NaCl, PMSF and 0.02% ß-mercaptoethanol. The His-tag was cleaved by overnight incubation with TEV protease at 4 °C and passed through a second Ni-NTA resin to recover only cleaved PDK1_50-359_. After a final gel filtration chromatography step, the protein was concentrated to ~24 mg/ml. Crystals were grown at room temperature using the hanging drop method in 24-well plates by mixing 1.5 μl of protein solution added with 5 mM ATP and 1.5 μl of the reservoir solution (1.2 M sodium citrate, 0.1 M sodium HEPES, pH 7.8) in a similar way as described before (54). Soaking was performed overnight either with 2 to 5 mM valsartan or with 5 to 10 mM HYG8. Crystal samples were cryoprotected with mother liquor added with 35% glycerol and flash-cooled in liquid nitrogen using Hampton Research loops (Aliso Viejo, California, USA). X-ray diffraction data were collected at the PROXIMA-2A beamline (Synchrotron SOLEIL, France) at 100 K using an EIGER X 9M detector. The structure of PDK1 (PDB code 3HRC (*54)*) served as a model for molecular replacement. Data were processed using Phaser, PHENIX and Coot (71–73) as previously reported (29). The crystals soaked with both 2 and 5 mM valsartan showed clear and unambiguous electron density corresponding to three copies of the ligand at the PIFpocket. No electron density corresponding to HYG8 was found in the crystals soaked with this ligand. Molecular graphic figures were prepared using PyMOL (Schrödinger). The refined structure was deposited with the accession code 8DQT.

### Small-angle X-ray scattering (SAXS)

SAXS data were collected at the P12 beamline (EMBL Hamburg, Germany) (74) using a Pilatus 6M detector (Dectris). Protein samples were measured in buffer containing 20 mM Tris-HCl pH 7,4, 250 mM NaCl, 1 mM DTT. In-line SEC-SAXS was performed using a Superdex increase 200 5/150 column (GE Healthcare) with a flow rate of 0.3 mL min^-1^ at room temperature. Acquired data were averaged and subtracted from an appropriate solvent blank to produce the final curve using the ATSAS Suite, EMBL (75) and CHROMIXS (76). Initial data pre-processing and reduction were performed using an automatic pipeline. Final scattering curves were analysed using PRIMUS for evaluation of molecular dimensions (Rg) (77) and maximum particle dimension (D_max_) using GNOM (78). The molecular mass was also estimated using Bayesian inference approach (79) and Volume-of-correlation (80). Ab initio models were computed with DAMMIF (81). SREFLEX (76) was employed for PDK1 catalytic domain to improve the agreement of flexible models to the experimental data. Finally, the flexibility of multidomain complexes for apo PDK1 FL and PDK1 FL + HYG8 was assessed with Ensemble Optimization Method 2.0 (56). The fit of the models to experimental data was assessed using CRYSOL (82) and superimpositions performed with Chimera and SASpy (83). The SASBDB accession codes for the SAXS projects are SASDNJ5 for SEC-SAXS PDK1_1-556_ and SASDNK5 for SEC-SAXS PDK1_1-556_ + HYG8. At the SAXS beamline P12, we measured 20 individual frames of sample and 40 frames of buffer, all under constant flow. The data thus corresponds to 20 cates (repetitions).

### Hydrogen/deuterium exchange experiments

The experiments were performed essentially as previously described for studies on the catalytic domain of PDK1 (84). In short, His-PDK1_1-556_ and His-PDK1_50-359_ were diluted in buffer containing deuterium oxide ([^2^H]_2_O) at 30°C for the indicated times. The exchange was stopped by quenching with acid ((0.5% TFA, 5 M GnCl) to achieve a final pH of 2.5) and cooling to 0°C. Each sample was immediately injected into nanoUPLC HDX Sample Manager (Waters) for online pepsin digestion using Poroszyme pre-packed pepsin column (Thermo Fisher Scientific), using 0.1% formic acid in LC-MS water at 100 μl/min, then trapped and desalted using a VanGuard C-18 column (Waters), followed by reverse-phase separation using ACQUITY™ 2.1 X 5 mm BEH C-18 column (Waters) using a 0.1% formic acid in acetonitrile gradient. The labelled derivative peptides were analyzed by Synapt G2 Si mass spectrometer (Waters) operating in positive ion mode using an MSE acquisition method. The mass spectrometer was continuously calibrated using 200 fmol/μl Glu-fibrinopeptide B standard flowing at 1 μl/min. The graphics represent the number of deuterium atoms incorporated into each peptide during the incubation.

### Molecular modeling

Atomic coordinates for starting structures were acquired from the Protein Data Bank (85): 1W1G was used for the PDK1 PH domain (39) and 4RRV (31) was used for the PDK1 catalytic domain. Molecular dynamics (MD) simulations of the PH domain bound to PIP_3_ were carried out with pmemd.cuda from AMBER18 (86–88) using AMBER ff14SB force field (89) and generalized amber force field (GAFF) (90). We used tLeap binary (part of AMBER18) for solvating structures in a cubed TIP3P water box with a 10 Å distance from structure surface to the box edges and closeness parameter of 0.75 Å. The system was neutralized and solvated. Simulations were carried out after minimizing the system, gradually heating the system from 0 K to 300 K over 50 ps and equilibrating the system for 1 ns at NPT. 500 ns of production was carried out using NPT at 300 K with the Langevin thermostat, a non-bonded interaction cut off of 8 Å, time step of 2 fs, and the SHAKE algorithm to constrain all bonds involving hydrogens. RMSD calculations were done with VMD (91) to analyze fluctuations in the structural ensemble to determine stability; stabilized regions close to the linker were then docked to the catalytic domain using ClusPro 2.0 (92–94) to form initial configurations of full-length PDK1.

Molecular dynamics simulations of the PH domain bound to PIP_3_ showed allosteric changes in stability in regions close to the linker region, namely in the helix at 434-443. This helix was then used for docking to the catalytic domain using ClusPro2.0 (92, 93) to give an orientation for the PH-catalytic domain interface. The helix at position 372-381 was modeled from the helix of the pseudosubstrate inhibitor peptide PKI (5-24) TTYADFIASGRTGRR bound to PKA from 1CDK (57), with Tyr^7^ and Phe^10^ matching with Tyr^373^ and Tyr^376^, respectively. The homology model of the helix was constructed by making point mutations using PyMOL. The helix at position 395-400 was docked to the catalytic domain with ClusPro2.0 and the remainder of the linker was built using the Builder in Pymol. The model was minimized and equilibrated using MD simulations similar to the above but using the implicit solvent forcefield GBNeck2 (95) igb=8 with PBRadii set to mbondi3. Finally, we simulated this model for five hundred nanoseconds and the PH domain stayed in the same pocket with both flanking helices 372-381 and 395-400 remaining bound to the catalytic domain. This points to having found a robust binding mode because weak docked poses will often leave their binding site during simulation.

### Stochastic optical reconstruction microscopy (STORM)-based method to analyze the proportion of PDK1 monomers and dimers

SNAP-PDK1_50-556_ (15 μM, in buffer Tris-HCl 50 mM pH 7.4, 200 mM NaCl, 200 mM imidazole and 1 mM DTT) was incubated with SNAP-Cell TMR-Star substrate (NEB) (30 μM) in the dark at room temperature for 1 h. Labelled SNAP-PDK1 was isolated by gel filtration using a PD MidiTrap G-25 Sephadex Column (GE). 10 μl labelled TMR-SNAP-PDK1 was diluted in buffer 50 mM Tris-HCl 50 mM pH 7.4 containing 100 mM NaCl and 1 mM DTT (control) or in the same buffer containing 15 μM HYG8 in a final volume of 20 μl. 1 μl of the mix was immediately diluted 200 fold in the same buffer and then added to a Lab-Tek™ 8 chamber slide system precoated with the positively charged polymer poly-(diallyldimethylammonium) chloride (PDDA). After incubating the sample 15 s with the polymer, the supernatant was removed, and each well was washed three times with ultra-pure water to wash out weakly and unbound protein. PDK1 was diluted to ensure individual molecules were separated. The slides were analysed using stochastic optical reconstruction microscopy (STORM) technology. Fluorescence from monomers was differentiated from that from dimers by the intensity of the fluorescence signals. The density of bright spots ensured that the random double occupation of a spot was negligible. Statistical analysis was performed using Infostat software (http://www.infostat.com.ar) by first verifying a normal distribution of all pooled residuals (Shapiro-Wilks test) and homogeneity of variance (F test), and then performing a Student’s t test. P values < 0.05 were considered to be statistically significant. The experiment was repeated three times, each of which showed highly statistically significant differences.

The STORM microscope used was custom-built around an OlympusIX-73 inverted microscope, as described previously (96). A 532 nm 1.5 W laser (Laser Quantum Ventus 532) was used for fluorescence excitation of TMR-Star. Light was focussed in the back focal plane of an oil immersion objective, Olympus PlanApo 60NA 1.42, which also collected the emission in epifluorescence mode. Images were recorded by an EMCCD camera (Andor iXon3 897). The camera and lasers were controlled with custom software developed in the laboratory. STORM experiments were carried out under 5 mW laser power on the sample. A pre amplifier gain of 5.1 and an electron multiplier gain of 30 were used in the CCD camera. Sequences of 100 frames with 100 ms of exposure time were collected in at least 15 different positions of each sample. Subsequent data analysis and the rendering of the final super-resolved image were performed with ImageJ/Fiji software with ThunderSTORM plugins. After calibration, the parameters selected in the STORM’s software were: shape, two color 120X; view, single; horizontal readout rate, 5.0 MHz; speed, 3.3 μs; clock voltage, normal; frame transfer mode, (select); set exposure time, 100 ms, real exposure time, 100 ms; real accumulation time, 102 ms; effective frame rate, 9.38 Hz; pre amplifier gain 5.1; electron multiplier gain, 30; laser power, 5 mW. An intensity histogram of bright spots was used to determine the amount of monomers and dimers in each sample. The image and processing of a representative region of a sample is presented in fig. S7A.

## Supplementary Material

Supplemental Material

## Figures and Tables

**Table 1:** Structural parameters and molecular mass determination obtained from SAXS Summary of structural parameters (Rg and Dmax ± error) obtained from SEC-SAXS, as well as the calculated molecular weight (MW) for each sample using different calculation approaches.

	Apo PDK1_50-359_	Apo PDK1_1-556_	PDK1_1-556_ + HYG8
	**SEC-SAXS**		
**Rg from Guinier (nm)**	2.37 (+/- 0.02)	3.48 (+/- 0.02)	3.50 (+/- 0.02)
**D_max_ from P(r) (nm)**	6.8 (+/- 0.3)	11.2 (+/-0.2)	11.5 (+/-0.1)
**Porod Volume Vp (nm^3^)**	59.1	107.5	98.1
**MW from Porod (kDa) (MW ~ Vp / 1.6)**	37	67	61
**MW Vc (kDa)**	35 (+/- 4)	74 (+/- 7)	54 (+/- 5)
**MW Bayesian (kDa)** *	39 (37-41; 90%)	79 (75-82); 90%	56 (53-59); 93%

* For Bayesian approximation, the MW estimate in kDa is indicated together with the credibility interval (kDa) and credibility interval probability (%) in parenthesis (calculated using PRIMUS).

## Data Availability

The refined structure for PDK1_50-359_ and valsartan was deposited in the Protein Databank with the accession code 7RLQ. The SASBDB accession codes are SASDNJ5 for SEC-SAXS PDK1_1-556_ and SASDNK5 for SEC-SAXS PDK1_1-556_ + HYG8. All other data needed to evaluate the conclusions in the paper are present in the paper or the Supplementary Materials. The pGEX-6P1 plasmid expressing the PDK1 PH domain (residues 408-556) is available from Dario Alessi under a material transfer agreement with MRC Protein Phosphorylation and Ubiquitylation Unit, UK.
