# Supplementary material for: Modulation of the substrate specificity of the kinase PDK1 by distinct conformations of the full-length protein
Source: Sci Signal. Author manuscript; Available in PMC 2023 Jun 22. (PMC7614687; doi:10.1126/scisignal.add3184)
Supplement: Supplemental Material [file EMS177231-supplement-Supplemental_Material.pdf]

SUPPLEMENTAL MATERIAL

Materials and Methods

Syntheses of HYG6 and HYG14

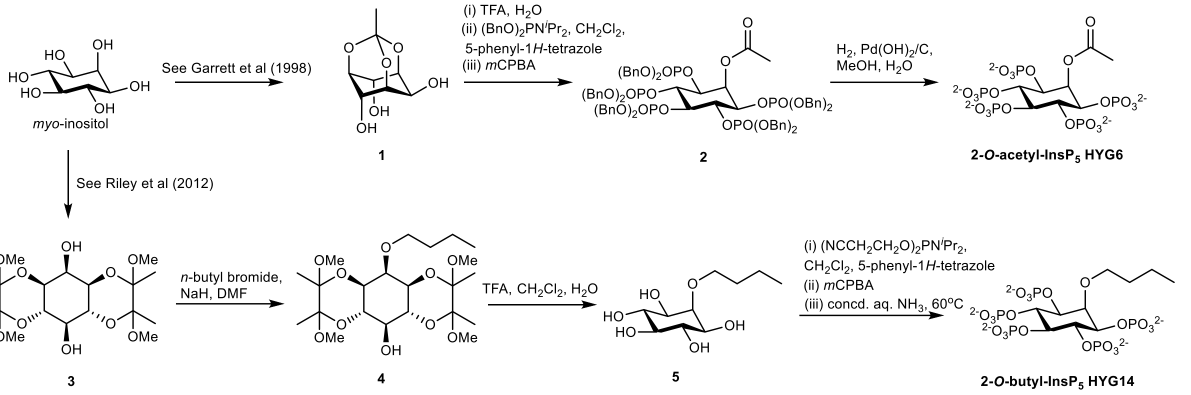

HYG6 (2-*O*-acetyl-IP$_5$) was synthesized from *myo*-inositol by way of *myo*-inositol orthoacetate (compound 1) (*99*) Thus, acid-catalyzed regioselective hydrolysis of compound 1 yielded crude 2-*O*-acetyl-*myo*-inositol, which was converted into full-protected pentakisphosphate (compound 2) by reaction with bis(benzyloxy)(*N*,*N*-diisopropylamino)phosphine activated with 5-phenyl-1*H*-tetrazole, followed by oxidation with *tert*-butyl hydroperoxide. Removal of the ten benzyl protecting groups by hydrogenolysis over Pd(OH)$_2$ on carbon yielded 2-*O*-acetyl-IP$_5$ (HYG6), which was purified by gradient elution anion exchange chromatography on Q Sepharose Fast Flow resin and isolated as the triethylammonium salt.

HYG14 (2-*O*-butyl-IP$_5$) was synthesized by way of diol (compound 3) (*100*). Thus, regioselective 2-*O*-alkylation of compound 3 with sodium hydride and 1-bromobutane in DMF yielded 2-*O*-butyl ether (compound 4). The acid-labile butanediacetal protecting groups were removed using aqueous trifluoroacetic acid to yield pentaol (compound 5) as a crystalline solid. Phosphitylation using bis(cyanoethyl)(*N*,*N*-diisopropylamino)phosphine, activated with 5-phenyl-1*H*-tetrazole, followed by oxidation with *m*CPBA, yielded the fully protected pentakisphosphate, from which the cyanoethyl protecting groups on phosphates were cleaved by β-elimination using aqueous ammonia at 60°C. The product was purified by gradient elution anion exchange chromatography on Q Sepharose Fast Flow resin to yield pure HYG14 isolated as the triethylammonium salt.

2-*O*-Acetyl 1,3,4,5,6-pentakis-*O*-[bis(benzyloxy)phosphoryl] *myo*-inositol (compound 2)
A mixture of TFA (1.8 ml) and water (0.2 ml) was added to orthoacetate (compound 1) (200 mg, 0.98 mmol) and the resulting solution was stirred for 5 min, after which TLC (ethyl acetate) indicated the complete conversion of starting material (R_f 0.5) into a product (R_f 0.0). The reaction mixture was co-evaporated with water, then with dichloromethane in vacuo to obtain 2-*O*-acetyl *myo*-inositol (218 mg) as a white solid, which was dried under vacuum. 5-phenyl-1*H*-tetrazole (1.32 g, 9.00 mmol) and dry dichloromethane (5 mL) were added to this solid under an atmosphere of argon, which was followed by the addition of bis(benzyloxy)(*N*,*N*-diisopropylamino)phosphine (1.81 mL, 5.40 mmol). Stirring continued for 1 h at room temperature, after which TLC (1:1, ethyl acetate:pet. ether) confirmed the complete consumption of starting material (R_f 0.0) to a product (R_f 0.9). The reaction mixture was cooled to –40 °C and 70% *t*BuOOH (1.29 mL, 9.00 mmol) was added portionwise while stirring. The cooling bath was removed and the mixture was allowed to reach room temperature. After 15 min, TLC (1:1, ethyl acetate:pet. ether) showed complete oxidation of pentakisphosphite to pentakisphosphate (R_f 0.3). The reaction mixture was diluted with dichloromethane (100 mL), washed with 10% sodium

sulphite solution (2 × 100 mL), dried and concentrated in vacuo. The residue was purified by column chromatography (pet. ether:ethyl acetate, 1:1 to 1:2) to generate Compound 2 (1.18 g, 80%) as a colorless oil. ^{31}P NMR (161.9 MHz, H-decoupled, $CDCl_3$) δ −1.84 (2 P, s), −1.46 (2 P, s), −1.21 (1 P, s, phosphate at C-5); ^{1}H NMR (400 MHz, $CDCl_3$) δ 2.00 (3H, s, CH_3), 4.43-4.54, 4.91-5.06 (3 H : 22 H, m, C-1-H, C-3-H, C-4-H, C-5-H, C-6-H and 10 × CH_2Ar), 6.00 (1 H, br s, C-2-H), 7.14-7.23 (50 H, m, Ar-H); ^{13}C NMR (100.6 MHz, $CDCl_3$) δ_C 20.6 (CO_2CH_3), 68.1 (C-2), 69.5, 69.6, 69.6, 69.7, 69.7, 69.8, 69.8 (10 × CH_2Ar), 72.9 (C-1 & C-3), 74.6 (C-5), 74.8 (C-4 & C-6), 127.9, 128.0, 128.0, 128.1, 128.1, 128.2, 128.2, 128.3, 128.3, 128.3, 128.4, 128.4, 128.4, 128.5 (50 × Ar-C), 135.6, 135.6, 135.8, 135.8, 135.8, 135.9 (10 × Ar-C_{ipso}), 169.0 (CO_2CH_3); HRMS (ESI-TOF) *m/z*: $[M + H]^+$ calculated for $C_{78}H_{80}O_{22}P_5$ 1523.3829; found 1523.3824; HRMS (ESI-TOF) *m/z*: $[M + Na]^+$ calculated for $C_{78}H_{79}O_{22}P_5Na$ 1545.3649; found 1545.3643.

2-*O*-Acetyl *myo*-inositol 1,3,4,5,6-pentakisphosphate (HYG6)

Compound 2 (600 mg, 0.39 mmol) was dissolved in methanol (15 mL) and water (3 mL) and 10% palladium hydroxide on activated charcoal (100 mg) was added. The resulting suspension was shaken in a Parr hydrogenator under H_2 for 20 h at room temperature. The catalyst was filtered through a PTFE syringe filter and the filtrate was evaporated under reduced pressure. The hygroscopic white foam was purified by ion exchange chromatography on Q Sepharose Fast Flow resin and eluted with a gradient of aqueous TEAB (0 to 2.0 M) to generate HYG6 (312 mg, 76%) as the triethylammonium salt. ^{31}P NMR (161.9 MHz, H-decoupled, D_2O) δ −0.75 (2 P, s), 0.24 (2 P, s), 0.57 (1 P, s, phosphate at C-5); ^{1}H NMR (400 MHz, D_2O) δ 1.12 (~37 H, t, *J* 7.4 Hz, CH_3 of TEA^+), 2.06 (3 H, s, CO_2CH_3), 3.05 (~25 H, q, *J* 7.4 Hz, CH_2 of TEA^+), 4.12-4.22 (3 H, m, *J* 2.8, 9.4, 9.8 Hz, C-1-H, C-3-H and C-5-H), 4.36 (2 H, ap. quartet, ddd, C-4-H and C-6-H), 5.58 (1 H, t, *J* 2.8 Hz, C-2-H); ^{13}C NMR (100.6 MHz, D_2O) δ_C 8.2 (CH_3 of TEA^+), 20.2 (CO_2CH_3), 46.5 (CH_2 of TEA^+), 71.6 (C-2), 72.1 (C-1 and C-3), 76.3 (C-4 and C-6), 77.0 (C-5), 172.6 (CO_2CH_3); HRMS (ESI-TOF) *m/z*: $[M - H]^-$ calculated for $C_8H_{18}O_{22}P_5$ 620.8983; found 620.9000.

2-*O*-Butyl 1,6:3,4-*bis*-[*O*-(2,3-dimethoxybutane-2,3-diyl)]-*myo*-inositol (compound 4)

Sodium hydride (118 mg of a 60% dispersion in oil, 2.94 mmol) was added in portions to a suspension of 1,6:3,4-bis-[*O*-(2,3-dimethoxybutane-2,3-diyl)]-*myo*-inositol (compound 3) (1.0 g, 2.45 mmol) in anhydrous DMF (60 ml). The resulting suspension was stirred for 1 h and 1-bromobutane (0.29 mL, 2.69 mmol) was added dropwise over 1 h. Stirring was continued for a further 20 h, after which TLC (hexane:ethyl acetate, 2:1) showed the conversion of starting material (R_f 0.0) to a product (R_f 0.3) and the excess sodium hydride was destroyed by the dropwise addition of methanol. The solvents were removed under reduced pressure and the residue was dissolved in dichloromethane (100 mL), washed with water (100 mL), brine (100 mL), dried ($MgSO_4$) and evaporated in vacuo. The resulting compound was purified by flash column chromatography (hexane:ethyl acetate, 2:1) to generate 2-*O*-butyl ether (compound 4) (870 mg, 77%) as a white solid, m.p. 185-187 °C (ethyl acetate); ^{1}H NMR (400 MHz, D_2O) δ 0.90 (3H, t, *J* = 7.4 Hz, $O(CH_2)_3CH_3$), 1.27 (6H, s, 2 x CH_3), 1.29 (6H, s, 2 x CH_3), 1.40 (2H, sextet, *J* = 7.4 Hz, $O(CH_2)_2CH_2CH_3$), 1.55 (2H, pentet, *J* = 6.1 Hz, $OCH_2CH_2CH_2CH_3$), 2.55 (1H, d, *J* = 2.0 Hz, 5-OH), 3.22 (6H, s, 2 x OCH_3), 3.26 (6H, s, 2 x OCH_3), 3.45 (2H, dd, $J_{1,2}$ = $J_{2,3}$ = 2.5 Hz, $J_{1,6}$ = $J_{3,4}$ = 10.2 Hz, C-1-H and C-3-H), 3.62-3.65 (2H, m, C-2-H and C-5-H), 3.70 (2H, t, *J* = 6.1 Hz, $OCH_2(CH_2)_2CH_3$), 3.97 (2H, t, $J_{4,5}$ = $J_{5,6}$ = 10.2 Hz, C-4-H and C-6-H); ^{13}C NMR (100.6 MHz, D_2O) δ_C 13.9 (q, $O(CH_2)_3CH_3$), 17.6 (q, 2 x CH_3), 17.7 (q, 2 x CH_3), 19.1 (t, $O(CH_2)_2CH_2CH_3$), 32.1 (t, $OCH_2CH_2CH_2CH_3$), 47.8 (q, 2 x OCH_3), 47.9 (q, 2 x OCH_3), 69.1 (d, C-1 and C-3), 69.3 (d, C-4 and C-6), 70.7 (d, C-5), 72.1 (t, $OCH_2(CH_2)_2CH_3$), 76.6 (d, C-2), 99.0 (s, $C(CH_3)OCH_3$), 99.5 (s, $C(CH_3)OCH_3$); elemental analysis calculated for $C_{22}H_{40}O_{10}$ (464.55) C, 56.88; H, 8.68; found C, 57.2; H, 8.79.

2-O-Butyl-*myo*-inositol (compound 5)

Aqueous TFA (90%, 5 mL) was added to a solution of 2-*O*-butyl ether (compound 4) (700 mg, 1.51 mmol) in dichloromethane (5 mL). The reaction mixture was stirred for 10 min at room

temperature, after which TLC (hexane:ethyl acetate, 2:1) indicated the complete conversion of starting material (R_f 0.3) into a product (R_f 0.0). The solvents were then removed by evaporation in vacuo followed by co-evaporation with methanol a few times until all the traces of butanedione (yellow in colour) was removed to give the pure tetraol (compound 5) (356 mg, quantitative) as a white solid, m.p. 224-226 °C (methanol); ^{1}H NMR (400 MHz, DMSO-d6) δ 0.87 (3 H, t, *J* 7.2 Hz, $O(CH_2)_3CH_3$), 1.32 (2 H, sextet, *J* 7.4 Hz, $O(CH_2)_2CH_2CH_3$), 1.45 (2 H, pentet, *J* 6.8 Hz, $OCH_2CH_2CH_2CH_3$), 2.88 (1 H, ddd, *J* 2.8, 4.3, 9.0 Hz, C-5-H), 3.14-3.19 (2 H, m, C-1-H and C-3-H), 3.32 (2 H, ddd, *J* 4.3, 9.0 Hz, C-4-H and C-6-H), 3.47 (1 H, t, *J* 2.6 Hz, C-2-H), 3.63 (2H, t, *J* 6.7 Hz, $OCH_2(CH_2)_2CH_3$), 4.37 (2 H, d, *J* 4.7 Hz, C-1-OH and C-3-OH), 4.55 (3 H, br s, C-4-OH, C-5-OH and C-6-OH); ^{13}C NMR (100.6 MHz, DMSO-d6) δ_C 13.9 ($O(CH_2)_3CH_3$), 18.8 ($O(CH_2)_2CH_2CH_3$), 32.1 ($OCH_2CH_2CH_2CH_3$), 72.0 (C-1 and C-3), 72.3 ($OCH_2(CH_2)_2CH_3$), 73.0 (C-4 and C-6), 75.3 (C-5), 81.8 (C-2); elemental analysis, calculated for $C_{10}H_{20}O_6$ (236.26) C, 50.84; H, 8.53; found C, 50.97; H, 8.61.

2-*O*-Butyl-*myo*-inositol-1,3,4,5,6-pentakisphosphate (HYG14)

To a solution of 2-*O*-butyl *myo*-inositol (compound 5) (300 mg, 1.27 mmol) and 5-phenyl-1*H*-tetrazole (1.39 mg, 9.52 mmol) in dry dichloromethane (10 mL) under an atmosphere of argon, was added bis(cyanoethyloxy)(*N*,*N*-diisopropylamino)phosphine (2.54 g, 9.52 mmol). Stirring was continued for 1 h at room temperature, after which TLC (ethyl acetate:ethanol, 4:1) confirmed the complete conversion of starting material (R_f 0.0) into a product (R_f 0.8). The reaction mixture was cooled to −40 °C and *m*CPBA (77%, 2.84 g, 12.70 mmol) was added portionwise while stirring. The cooling bath was removed and the mixture was allowed to reach room temperature. After 15 min, TLC (ethyl acetate:ethanol, 4:1) showed complete oxidation of pentakisphosphite to pentakisphosphate (R_f 0.2) and the reaction mixture was diluted with ethyl acetate (100 mL), washed with 10% sodium sulphite solution (2 × 200 mL), dried and solvent evaporated in vacuo. The resulting compound was then dissolved in concentrated aqueous ammonia solution (30 mL) and heated at 60°C overnight in a Pyrex pressure tube. After evaporation of solution under vacuum, the residue was purified by ion exchange chromatography on Q Sepharose Fast Flow resin and eluted with a gradient of aqueous TEAB (0 to 2.0 M) to yield the pure triethylammonium salt of 2-*O*-butyl *myo*-inositol 1,3,4,5,6-pentakisphosphate HYG14 (1.12 g, 81%) as a hygroscopic white solid. ^{31}P NMR (161.9 MHz, H-decoupled, D_2O) δ −0.70 (2P, s), 0.11 (2P, s), 0.65 (1P, s, phosphate at C-5); ^{1}H NMR (400 MHz, D_2O) δ 0.68-0.73 (3H, m, $O(CH_2)_3CH_3$), 1.05-1.10 (~34H, m, CH_3 of TEA^+), 1.20-1.23 (2H, m, $O(CH_2)_2CH_2CH_3$), 1.38-1.40 (2H, m, $OCH_2CH_2CH_2CH_3$), 2.96-3.02 (~24H, m, CH_2 of TEA^+), 3.65-3.67 (2H, m, $OCH_2(CH_2)_2CH_3$), 3.96-4.03 (4H, m, C-1-H, C-2-H, C-3-H and C-5-H), 4.26-4.31 (2H, m, C-4-H and C-6-H); ^{13}C NMR (100.6 MHz, D_2O) δ_C 8.1 (q, CH_3 of TEA^+), 13.2 (q, $O(CH_2)_3CH_3$), 18.5 (t, $O(CH_2)_2CH_2CH_3$), 31.4 (t, $OCH_2CH_2CH_2CH_3$), 46.5 (t, CH_2 of TEA^+), 73.9 (t, $OCH_2(CH_2)_2CH_3$), 74.1 (m, C-1 and C-3), 76.3 (m, C-4 and C-6), 77.5, 78.0 (2 × m, C-2 and C-5); HRMS (ESI-TOF) *m/z*: $[M - H]^-$ calculated for $C_{10}H_{24}O_{21}P_5$ 634.9504; found 634.9525

Figure S1

A

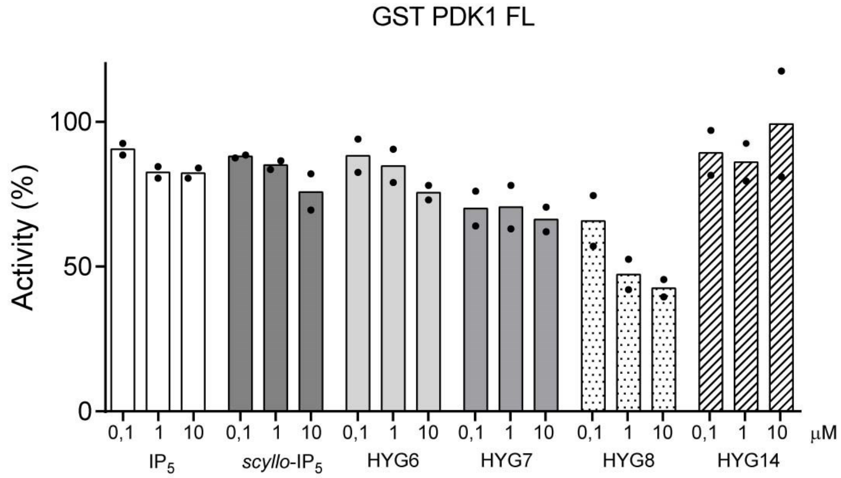

B

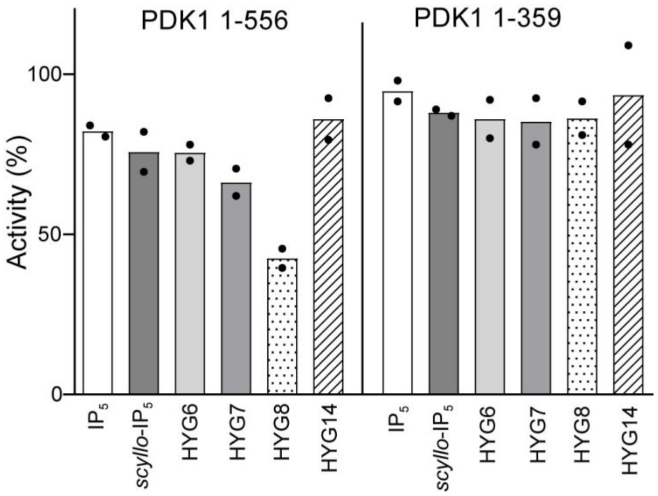

Figure S1. Inositol phosphates and derivatives do not potently inhibit the intrinsic activity of PDK1. (A) The effect of inositol phosphates and derivatives was assessed on the kinase activity of full-length GST-PDK1$_{1\text{-}556}$ toward T308tide. (B) The effect of inositol phosphates and derivatives (10μM) was assessed on the kinase activity of full-length GST-PDK1$_{1\text{-}556}$ and a construct comprising the catalytic domain but lacking the linker region and the PH domain, PDK1$_{1\text{-}359}$, toward T308tide. N=2 independent experiments.

Figure S2

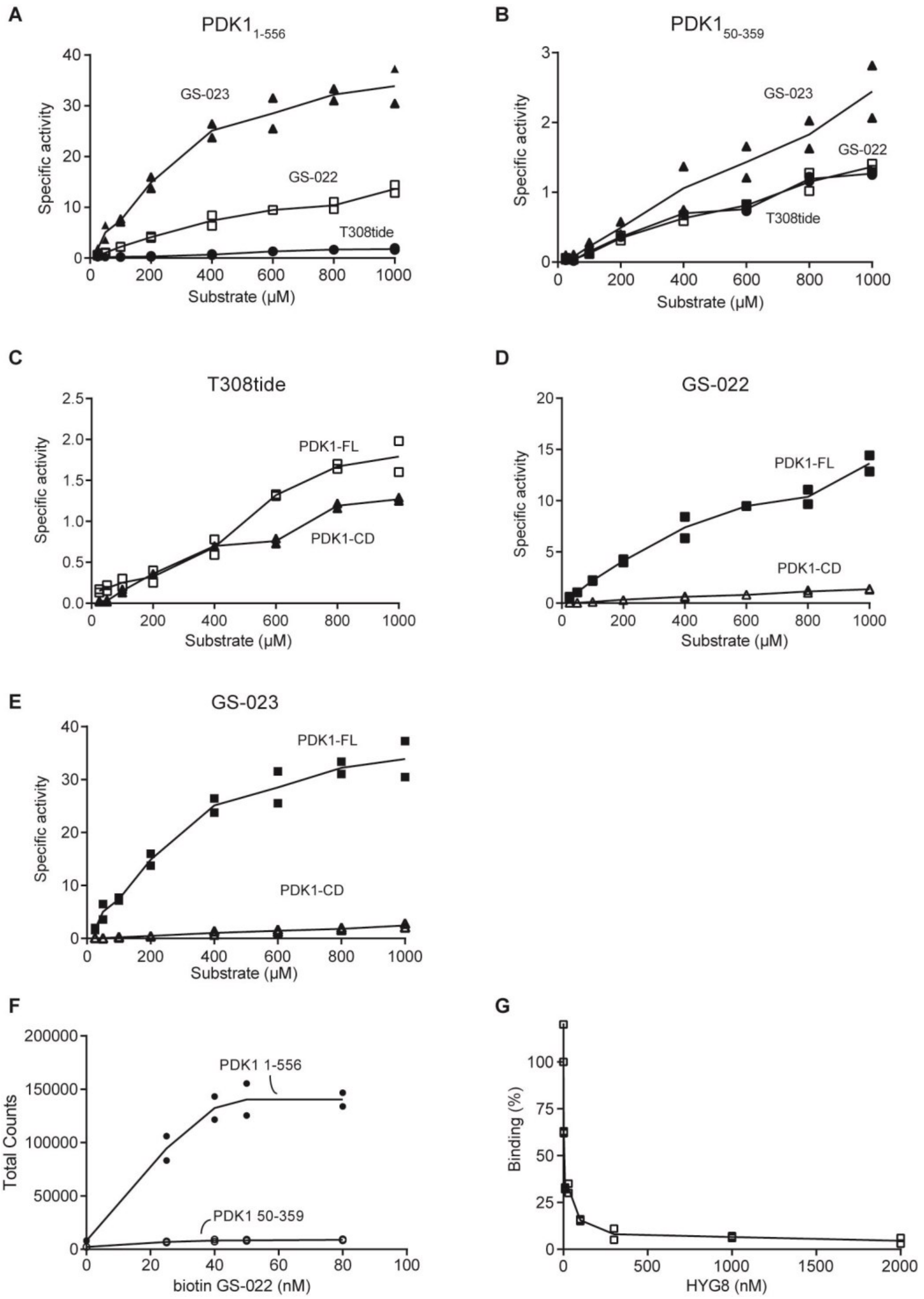

Figure S2. Analysis of the "PDK1 Direct" commercial assay. (A-E) The activity of GST-PDK1$_{1\text{-}556}$ or GST-PDK1$_{1\text{-}359}$ was measured using [γ^{32}P]ATP and different substrates: T308tide, derived from the activation loop of Akt and two alternative "improved" substrates; GS-022 (RRRQFSLRRKAK); and GS-023 (RRRQFSLRRKA-K(5-FAM)). (A) Specific activity of GST-PDK1$_{1\text{-}556}$ towards the three substrates. N=2 independent experiments. (B) Specific activity of GST-PDK1$_{1\text{-}359}$ towards the three substrates. N=2 independent experiments. (C) Analysis of T308tide as a substrate of GST-PDK1$_{1\text{-}556}$ and GST-PDK1$_{1\text{-}359}$. N=2 independent experiments. (D) Analysis of GS-022 as a substrate of GST-PDK1$_{1\text{-}556}$ and GST-PDK1$_{1\text{-}359}$. (E) Analysis of GS-023 as a substrate of GST-PDK1$_{1\text{-}556}$ and GST-PDK1$_{1\text{-}359}$. N=2

independent experiments. (F-G) Interaction and displacement of interactions using AlphaScreen technlology. (F) Interaction of biotin-GS-022 with GST-PDK1$_{1-556}$ and GST-PDK1$_{1-359.}$ N=2 independent experiments. (G) Effect of HYG8 on the interaction between biotin-GS-022 and GST-PDK1$_{1-556.}$ N=2 independent experiments.

The Thermo Fisher Scientific “PDK1 Direct” assay produced results quite different from those obtained using our PDK1 kinase assay which uses [γ^{32}P]ATP and T308tide (KTFCGTPEYLAPEVRR) as substrates. Phosphorylation of T308tide and of the alternative peptides GS-022 (RRRQFSLRRKAK) and GS-023 (RRRQFSLRRKA-K(5-FAM)) was compared. GS-023 and GS-022 were phosphorylated efficiently by GST-PDK1$_{1-556}$, with over 30 and 10-fold higher turnover than T308tide, respectively (fig. S2A). The increased phosphorylation of GS-023 indicated that the fluorescein molecule at the C-terminal of the GS-023 peptide provided a degree of improvement of substrate phosphorylation by PDK1$_{1-556}$. GST-PDK1$_{1-359}$, comprising only the catalytic domain but lacking the linker region and the PH domain, phosphorylated the three substrates similarly, with a slight increased turnover towards GS-023 (fig. S2B). Thus, the phosphorylation of GS-23 and GS-022, but not that of T308tide, depended on the construct used (figs. S2C-E). To assess a possible differential binding of GS-022 to GST-PDK1$_{1-556}$ and GST-PDK1$_{1-359}$, we used an AlphaScreen assay that measured the interaction of biotin-GS-022 with GST-PDK1$_{1-556}$ and GST-PDK1$_{1-359}$. Biotin-GS-022 showed increased binding to GST-PDK1$_{1-556}$ (150 nM) compared to the construct comprising the catalytic domain (fig. S2F). We concluded that the increased phosphorylation of GS-022 could be due to an additional interaction site for the linker-PH domain which increases the affinity of GS-022 for PDK1$_{1-556}$. HYG8 potently displaced the interaction between GS-022 and PDK1$_{1-556}$ (fig. S2G), which could explain how a decrease in the phosphorylation of a substrate in an in vitro protein kinase assay could be due to a compound altering the docking interaction of the peptide substrate. We suggest that the inhibitory effect of HYG8 in the "PDK1 Direct" assay could be due to the specific disruption of a "docking" interaction of the Ser/Thr 07 peptide substrate with PDK1. Although the “PDK1 direct” assay does not always reflect the intrinsic kinase activity of PDK1, our results indicate that the Thermo Fisher Scientific activity assays using Ser/Thr 07 or the AlphaScreen interaction between PDK1 and biotin-GS-022 can be used to assess the relative conformation of PDK1.

Figure S3

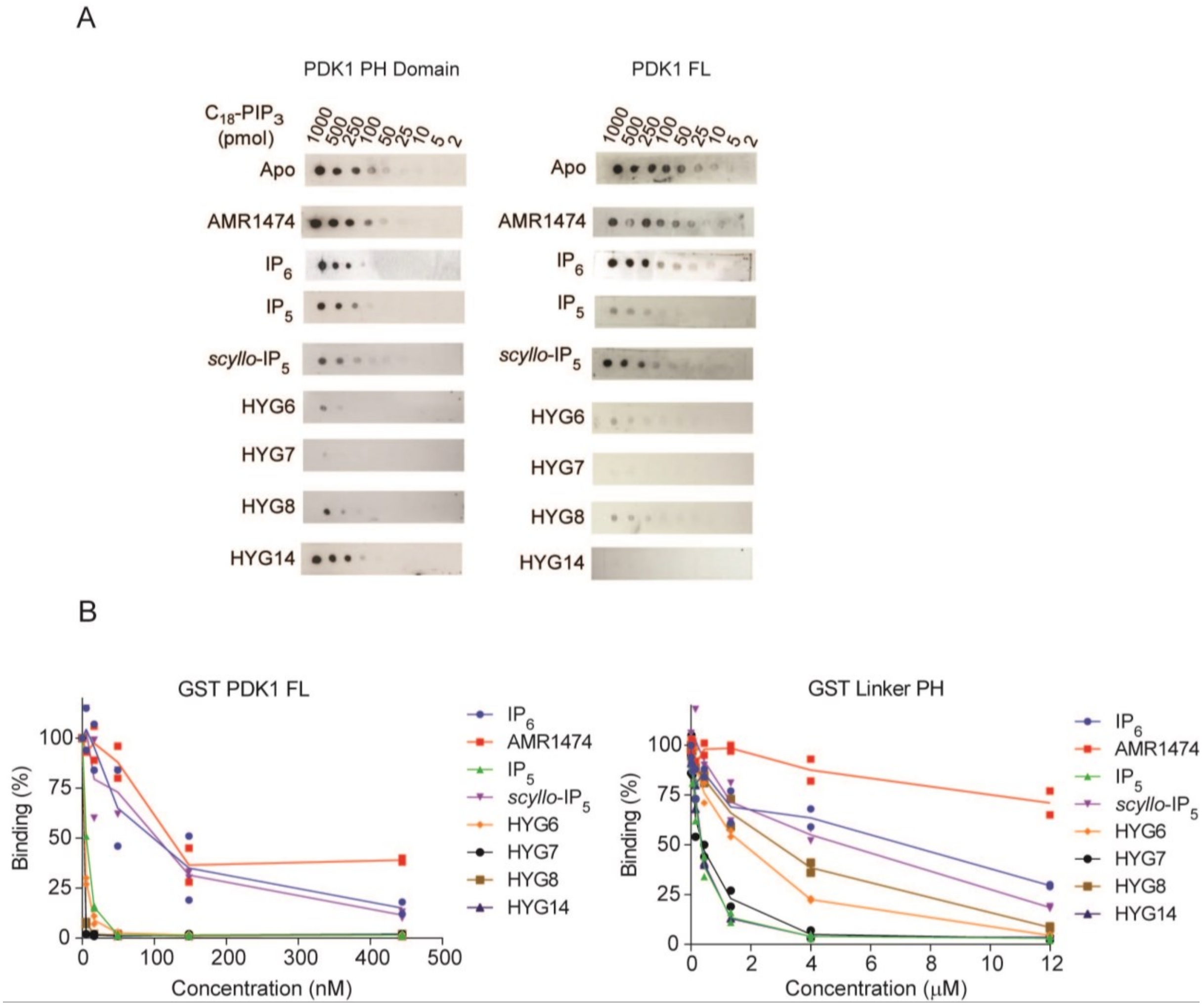

Figure S3. The ability of inositol phosphates and derivatives to displace PIP$_3$ from PDK1$_{1\text{-}556}$ and from the PH domain of PDK1 can depend on the construct used. (A) The effect of inositol polyphosphates and synthetic derivatives (10 µM and 1 µM respectively) on the interaction between the isolated PH domain of PDK1 (residues 408-556; 10 nM) or full-length PDK1 (GST-PDK1$_{1\text{-}556}$; 10 nM) and C$_{18}$-PIP$_3$ spotted on a membrane. N=2 independent experiments. (B) The interaction of linker-PH(PDK1$_{360\text{-}556}$)–PIP$_3$ and PDK1-FL (PDK1$_{1\text{-}556}$)–PIP$_3$ was measured using AlphaScreen technology and the ability of compounds to displace the interaction was tested. These are the expanded curves for all compounds of the results shown in Figs. 3D-G (N=2 independent experiments).

Figure S4

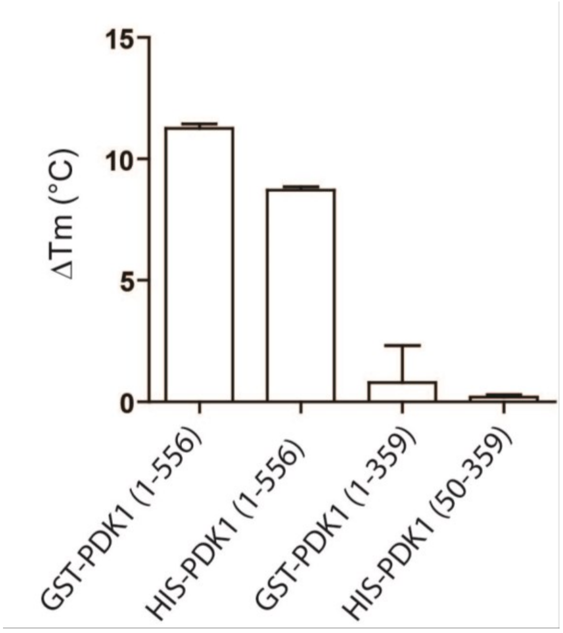

Figure S4. HYG8 stabilizes GST-PDK1$_{1\text{-}556}$ and His-PDK1$_{1\text{-}556}$ in thermal shift assays. The effect of HYG8 (20 μM) on the thermal stability of different constructs of PDK1 was assessed. N=3 independent experiments.

Figure S5

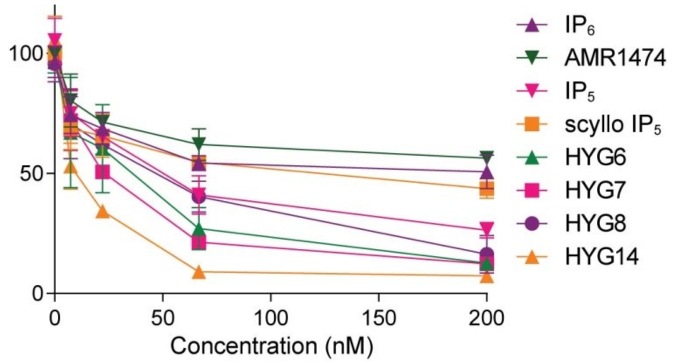

Figure S5. Displacement of PDK1 FL - PDK1 FL dimers by all compounds. The ability of compounds to displace the interaction between His-PDK1$_{1\text{-}556}$ and GST-PDK1$_{1\text{-}556}$ was measured using AlphaScreen technology. N=3 independent experiments. Fig. 4D presents a subset of these compounds for clarity.

Figure S6

A **B**

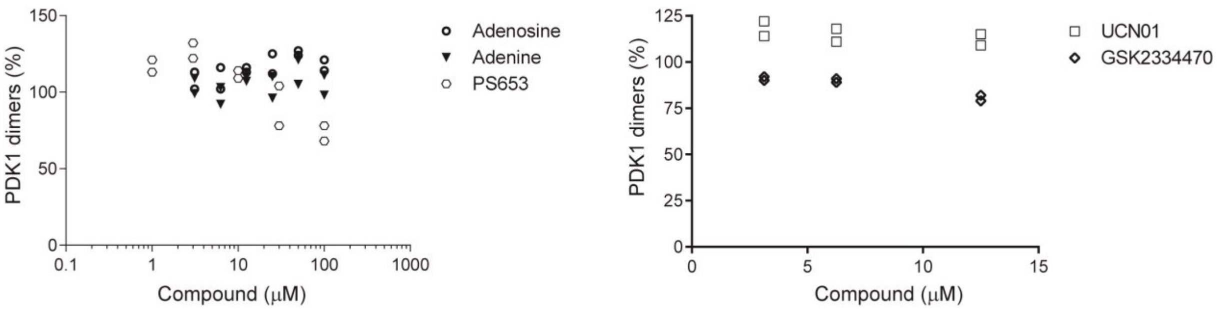

Figure S6. Small molecules that bind to the ATP-binding site and allosterically affect the PIF pocket do not affect the formation of PDK1 dimers.
(A-B) The effect of adenosine, adenine, PS653, GSK2334470 and UCN01 on the formation of His-PDK1$_{1\text{-}556}$ and GST-PDK1$_{360\text{-}556}$ dimers was evaluated using an AlphaScreen-based interaction assay. N=2 independent experiments.

Figure S7

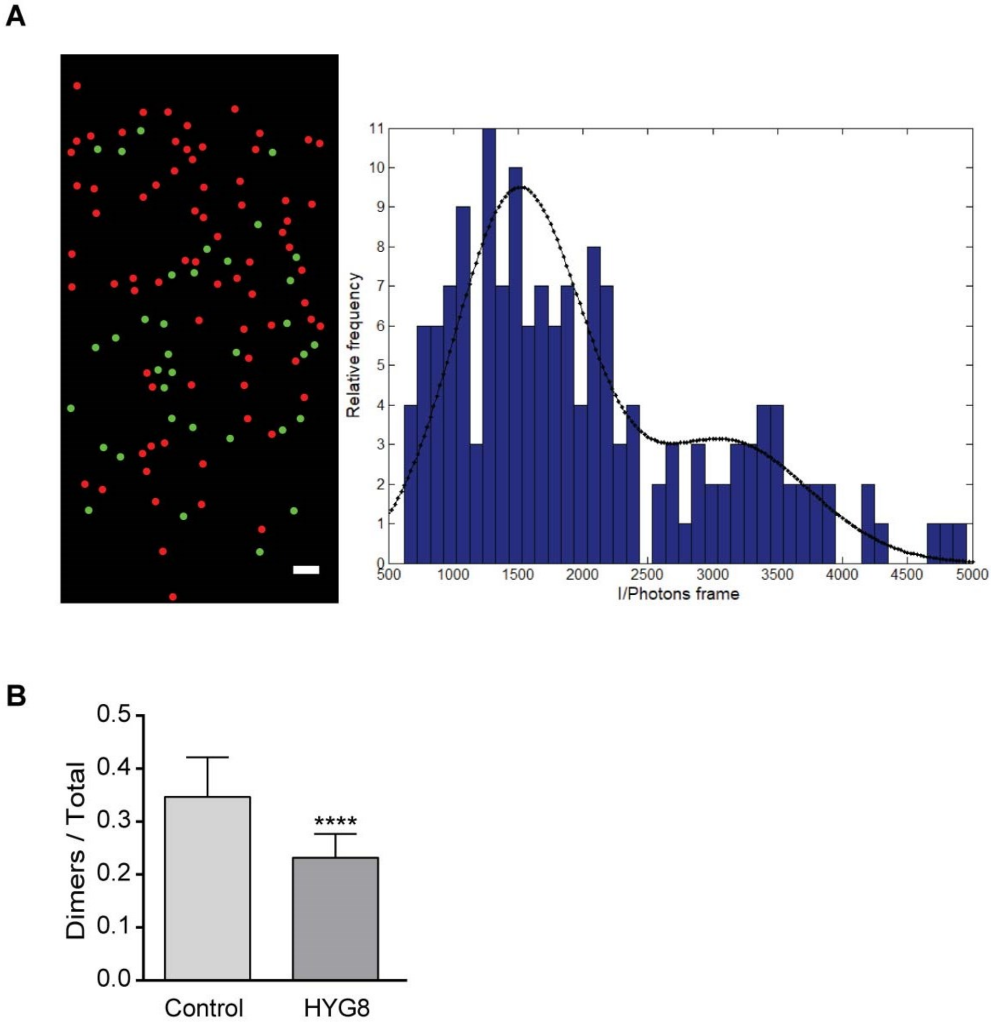

Figure S7. Single particle fluorescence identifies PDK1$_{1}$ dimers and disruption of dimers by HYG8. (A) STORM image with 100 ms exposure showing single molecule localizations of His-SNAP-PDK1$_{50\text{-}556}$ labelled with TMR. Monomers are shown as red dots (74 localizations displayed in the image), whereas dimers are shown as green dots (35 localizations in the image). An intensity histogram of the brightness of the spots is shown in the image, fitted to a sum of two Gaussians. Monomers are identified as spots in the intensity range 500-2500, whereas dimers correspond to the intensity range 2500-4500. For each independent experiment, 15 random images like the one shown were used to average the proportion of monomers and dimers under each condition. Scale bar 1.5µm. (B) Single particle fluorescence identifies PDK1$_{1\text{-}556}$ dimers and disruption of dimers by HYG8. Shown is one of three independent experiments (quantification for 15 sections each), which all showed highly significant differences according to a Student's t test (****, p<0.0001).

Figure S8

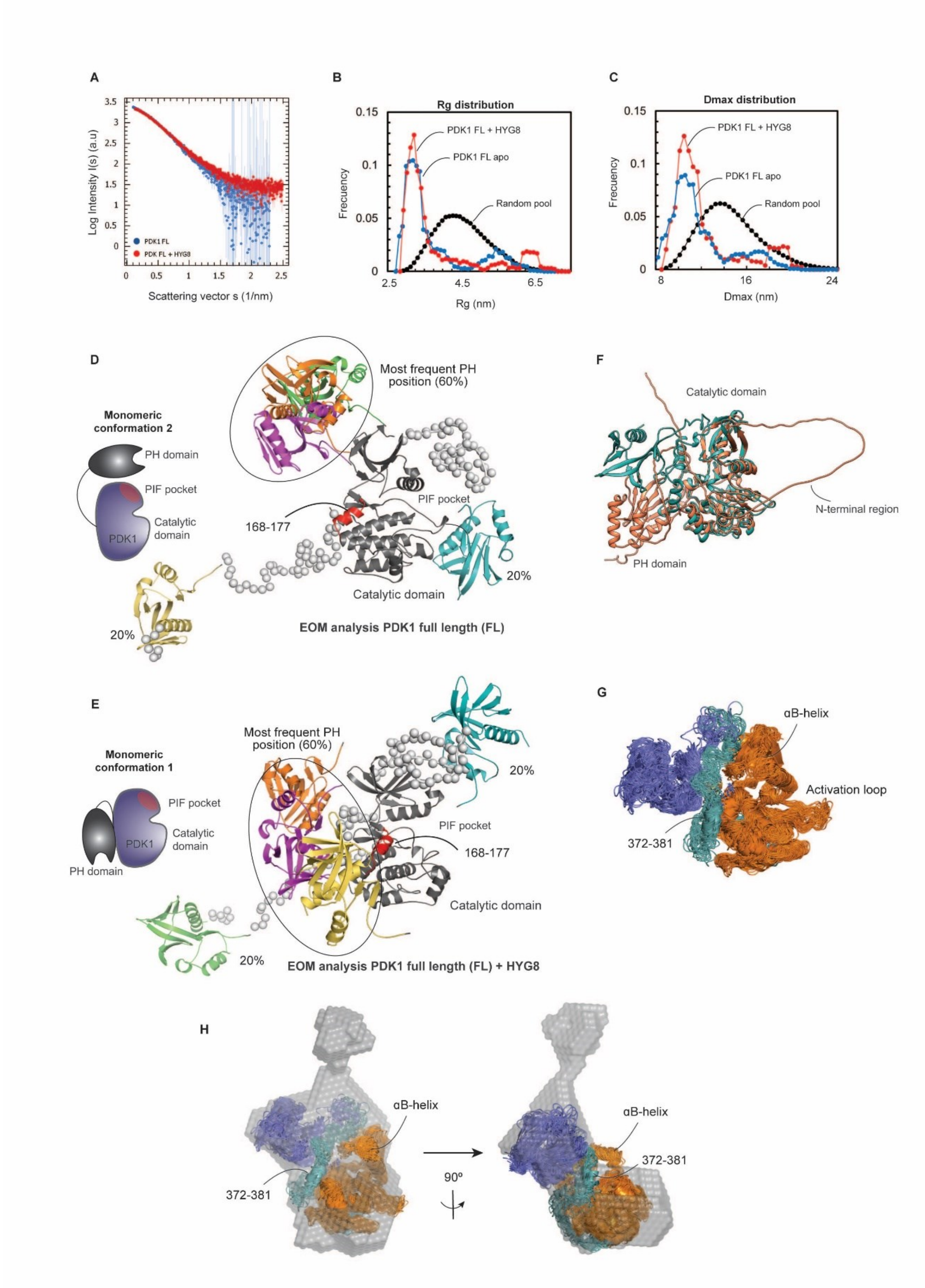

Figure S8. SEC-SAXS experiments of PDK1. (A) Scattering plot (obtained using CHROMIXS) corresponding to SEC-SAXS data from PDK1 FL in the absence and presence of HYG8. (B-C) EOM analysis of SEC-SAXS data from PDK1 full-length in the absence (blue) and presence (red) of HYG8. The “random pool” indicates the theoretical data that

would be expected if the position of the linker PH domain was fully flexible and randomly located in the structure. (D-E) Proposed EOM models for apo PDK1 full-length in the absence (D) and presence of HYG8 (E). Dummy atoms predicted for the linker and N-terminal region are only shown for one of the five EOM models to simplify the overall representation. The catalytic domain shown in E is slightly rotated to the right in comparison to D to achieve a clearer view of five alternative positions of the PH domain. (F) Proposed model for PDK1 full-length in the presence of HYG8 (see also Fig. 7A) in cyan aligned to the proposed model by AlphaFold (salmon). Both model structures protect the “back” of the catalytic domain. (G) Molecular dynamics (MD) of the proposed model for PDK1 in the presence of HYG8 (presented in Fig. 7A). (H) The MD of the modeled structure of PDK1 is shown fitted into the most representative ab initio low resolution structure DAMMIF (Fig. 4K) of His-PDK1$_{1\text{-}556}$ + HYG8 determined from experimental SAXS data (light grey).

Figure S9

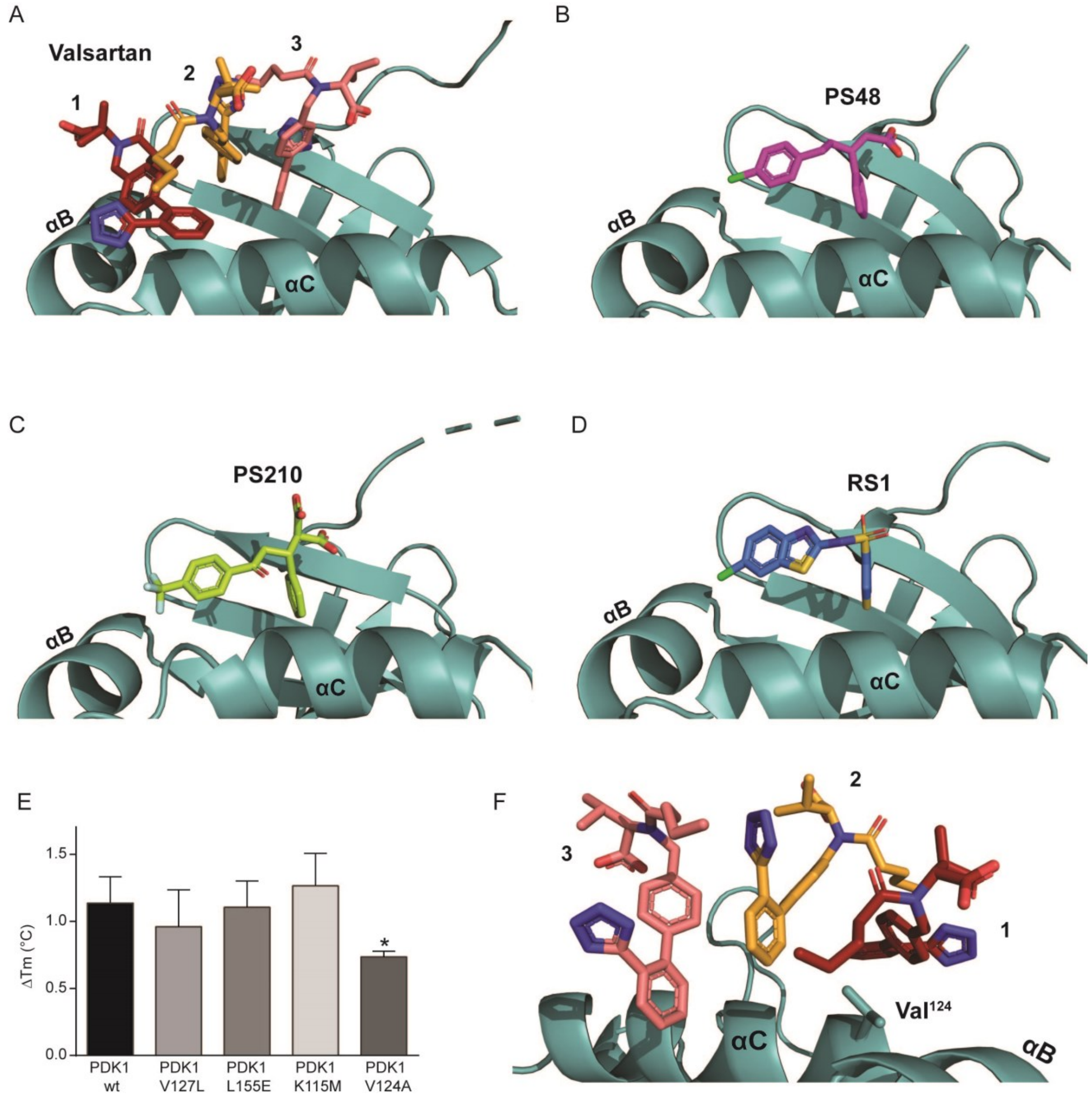

Figure S9. Structural comparison between the binding mode of valsartan and other small molecules that target the PIF pocket. (A) Structure of PDK1 CD ($PDK1_{50\text{-}359}$) in complex with valsartan. (B) PDK1 CD bound to PS48 (PDB: 3HRF, (*53*)). (C) PDK1 bound to PS210 (PDB: 4AW1, (*28*)). (D) PDK1 bound to RS1 (PDB: 4RQK, (*30*)). (E) Differential scanning fluorimetry of WT and PIF pocket mutants of $PDK1_{1\text{-}556}$ to determine the effect of valsartan (100 μM) on melting temperature (Tm). N=4 independent experiments for each group. * $p<0.05$ One-way ANOVA followed by Dunnet post-hoc multiple comparisons tests (PDK1 WT as the control condition). (F) Structure of PDK1 CD PIF pocket in complex with valsartan showing that Val^{124} interacts with valsartan molecule #1.

Figure S10

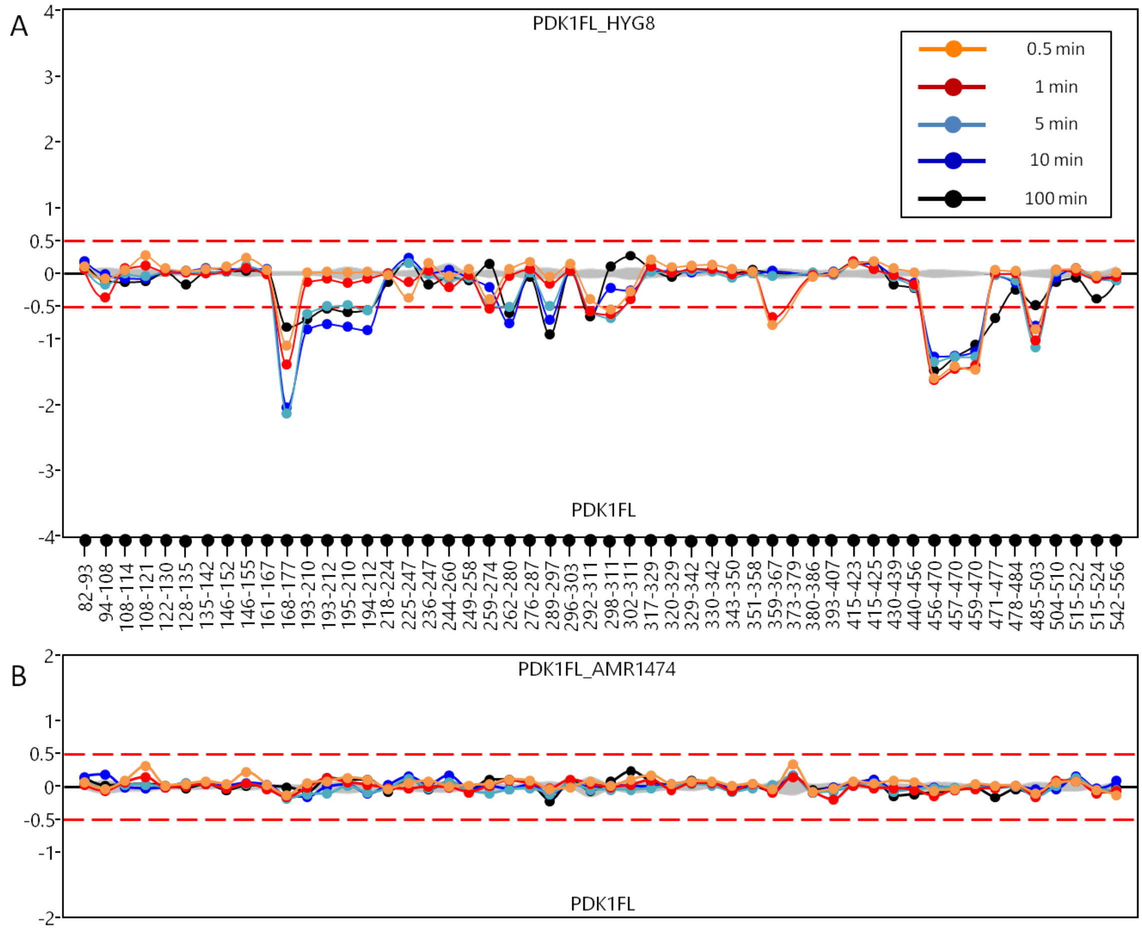

Figure S10. Hydrogen/deuterium exchange profiles. (A) The graph shows the difference in deuterium incorporation between PDK1 FL (PDK1$_{1\text{-}556}$) and PDK1 FL + HYG8 for each of the detected peptides. N=3 independent experiments. (B) The difference plot between PDK1 FL and PDK1 FL + AMR1474. N=3 independent experiments.

Figure S11

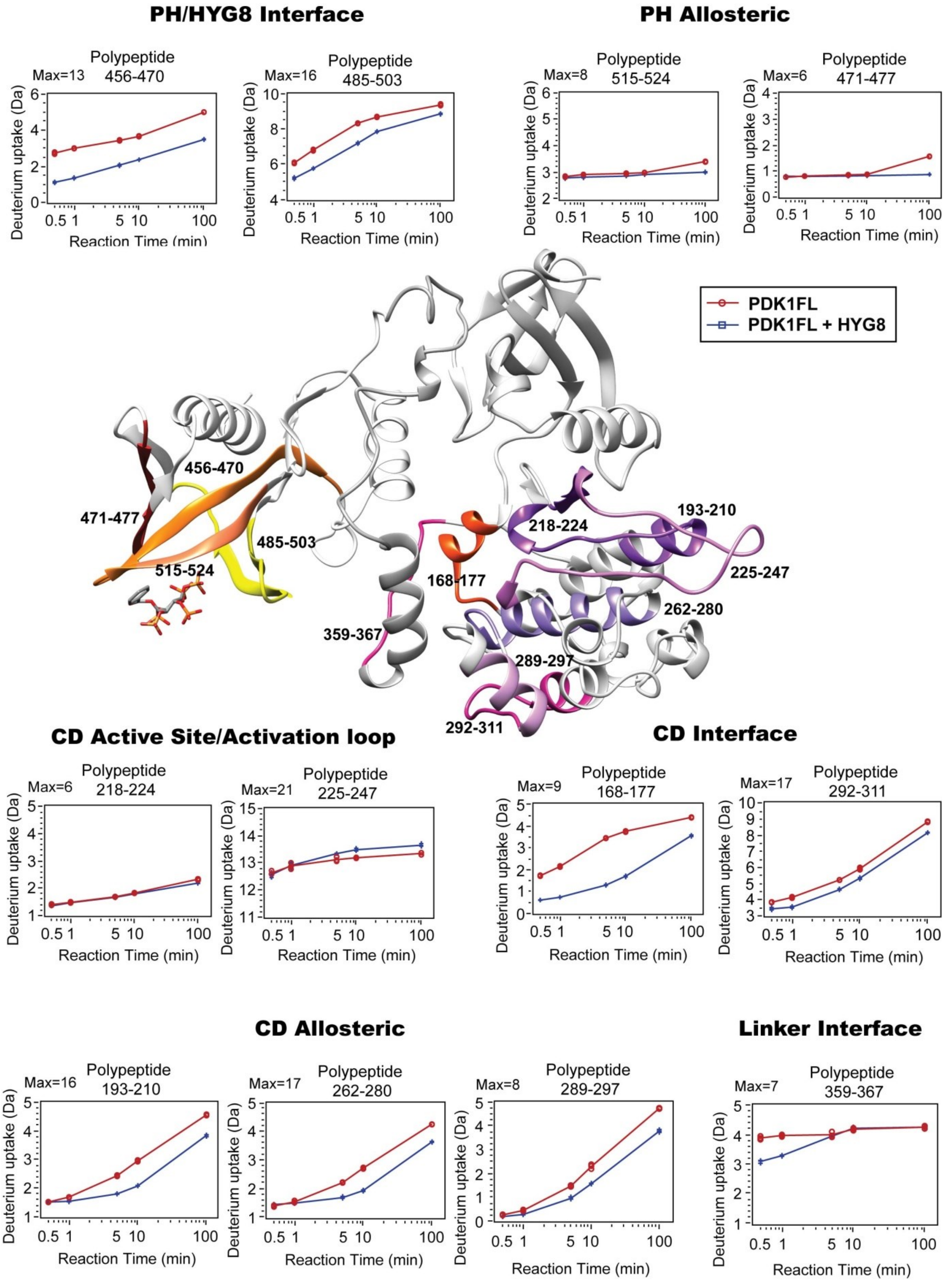

Figure S11. The H/D exchange results and the model of full-length PDK1 in the conformation stabilized by HYG8. The structural model of PDK1$_{77-549}$ is presented at the center, indicating polypeptides identified by H/D exchange. The inset graphics show the time course of the protection of the different polypeptides in the absence or presence of HYG8. The polypeptides are grouped according to the structural feature that explains the effect observed. N=3 independent experiments.

Figure S12

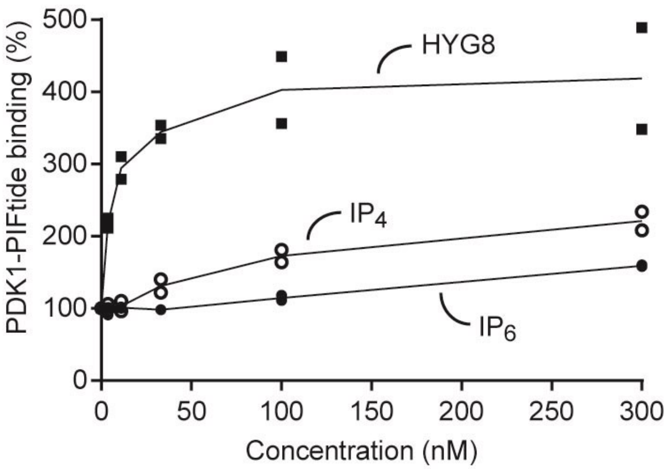

Figure S12. Effect of the PIP_3 head group on the interaction between PDK1 and PIFtide. AlphaScreen interaction assay between GST-$PDK1_{1\text{-}556}$ (1 nM) and biotin-PIFtide (5 nM) in the presence of HYG8, IP_4 or IP_6. N=2 independent experiments.

Table S1: Thermo Fisher/Invitrogen's SelectScreen® Profiling Service. IC_{50} was calculated from 10-point titration experiment in which each data point was obtained from N=2 independent experiments. Z′-factor is indicated as a measure of data quality.

Compound	Kinase Assay	IC_{50} (nM)	Hill slope	R^2 value	Z'
HYG8	Cascade	6.42	2.19	0.9810	0.62
IP_5	PDK1 direct	577	1.33	0.9921	0.78
HYG6	PDK1 direct	449	1.11	0.9950	0.78
HYG7	PDK1 direct	29	1.39	0.9994	0.78
HYG8	PDK1 direct	24.5	1.39	0.9985	0.78
HYG14	PDK1 direct	16.2	1.61	0.9958	0.82
IP_6	PDK1 direct	>>10,000	–2.18	0.3276	0.78

Table S2: Single point results from Thermo Fisher/Invitrogen's ¨PDK1 Direct¨: SelectScreen® Profiling Service. N=2 independent experiments. Z′-factor is indicated as a measure of data quality.

Compound Name	Kinase Assay	Inhibition at 1 µM (%)	% Inhibition		Z'
			Point 1	Point 2	
HYG6	PDK1 Direct	59	53	64	0.76
HYG7	PDK1 Direct	100	101	99	0.76
HYG8	PDK1 Direct	98	97	98	0.76
HYG14	PDK1 Direct	100	98	101	0.76
IP_5	PDK1 Direct	66	63	69	0.88
scyllo-IP_5	PDK1 Direct	-9	-8	-10	0.88
AMR1474	PDK1 Direct	10	9	11	0.88
IP_6	PDK1 Direct	8	8	7	0.80

Table S3. Temperature stabilization of $PDK1_{1\text{-}556}$ by inositol phosphates and derivatives (N=3 independent experiments).

	ΔTm (°C)	
**Compound**	**1 µM**	**20 µM**
IP_6	6.2	9.0
AMR1474	2.9	6.0
scyllo-IP_5	4.7	7.3
IP_5	5.3	11.9
HYG6	7.8	9.9
HYG7	12.6	14.5
HYG8	11.7	13.7
HYG14	15.2	16.5

Table S4. Displacement of His-PDK1$_{1-556}$ interaction with GST-PDK1$_{360-556}$ by inositol phosphates and derivatives in the absence or presence of PIFtide.

	PDK1 1-556 + PDK1 360-556			
			with PIFtide	
**Compound**	**IC50 (nM)**	**95% CI**	**IC50 (nM)**	**95% CI**
IP_6 *	-	-	-	-
AMR1474	458	225 to 934	159	12 to 2027
scyllo-IP_5	124.2	28 to 545	140	69 to 282
IP_5	67	28.3 to 158.6	59.4	44.6 to 79.1
HYG6	27	18 to 41	17.9	13.2 to 24.4
HYG7	22.6	20 to 25.7	22.7	18.9 to 27.3
HYG8	33.3	23.9 to 46.5	19.5	10.3 to 36.9
HYG14	29.6	24.7 to 35.4	29.4	16.2 to 53.3

* Does not reach complete displacement.

Table S5. Structural parameters and molecular mass determination obtained from Batch-SAXS

	**Apo PDK 1 CD**	**Apo PDK1 FL**	**PDK1 FL + HYG8**
	**BATCH-SAXS**		
**Rg from Guinier (nm)**	2.36 (± 0.01)	4.26 (± 0.04)	3.75 (± 0.02)
**Dmax from P(r) (nm)**	7.0 (± 0.3)	14.0 (± 0.2)	12.5 (± 0.4)
**Porod Volume Vp (nm^3)**	60.5	123.2	103.3
**MW from Porod (kDa) (MW ~ Vp / 1.6)**	38	77	65
**MW Vc (kDa)**	32 (± 4)	88 (± 8)	53 (± 5)
**MW Bayesian (kDa)**	32 (31-34; 96%)	94 (90-99; 96%)	53 (53-60; 96%)

Table S6. Data collection and refinement statistics for crystallography

	PDK1-CD-Valsartan
**Data collection**	
Wavelength (Å)	0.9801
Crystal-to-detector distance (mm)	116.54
Rotation range per image (°)	0.1
No. of frames	3600
Exposure time per image (s)	0.025
**Indexing and scaling**	
Space group	*C*2
Cell dimensions	
a, *b*, *c* (Å)	148.51, 44.59, 48.21
α,β,γ (°)	90, 101.32, 90
Resolution (Å)	36.51 – 1.31 (1.39 – 1.31)*
Mosaicity (°)	0.100
R_{meas}	0.055 (1.306)
$CC_{1/2}$ (%)	99.9 (74.4)
$I / \sigma(I)$	15.9 (1.0)
Completeness (%)	99.4 (97.4)
Redundancy	6.7 (6.6)
Solvent content (%)	45
Overall *B*-factor from Wilson plot (Å^2)	25
**Refinement**	
No. of reflections	73419
R_{work} / R_{free}	0.199 / 0.224
No. of atoms	
Protein	2354
Ligand/ion	110
Water	133
B-factors (Å^2)	
Protein	24
Ligand/ion	39
Water	29
Overall	25
R.m.s deviations (*101*)	
Bond lengths (Å)	0.009
Bond angles (°)	1.11
**MolProbity validation (*102*)**	
Clashscore	4.54
MolProbity Score	1.25
Ramachandran plot	
Favored (%)	97.9
Allowed (%)	2.1
Disallowed (%)	-
**Protein Data Bank deposition**	
PDB code	8DQT

*Values in parentheses are for the highest-resolution shell.
